# The PINK1/Parkin pathway of mitophagy exerts a protective effect during prion disease

**DOI:** 10.1371/journal.pone.0298095

**Published:** 2024-02-23

**Authors:** Anne Ward, Forrest Jessop, Robert Faris, Jason Hollister, Daniel Shoup, Brent Race, Catharine M. Bosio, Suzette A. Priola

**Affiliations:** 1 Laboratory of Neurological Infections and Immunity, Rocky Mountain Laboratories, National Institute of Allergy and Infectious Diseases, Hamilton, Montana; 2 Laboratory of Bacteriology, Rocky Mountain Laboratories, National Institute of Allergy and Infectious Diseases, Hamilton, Montana; 3 Department of Microbiology and Immunology, Carver College of Medicine, University of Iowa, Iowa City, Iowa, United States of America; University of Melbourne, AUSTRALIA

## Abstract

The PINK1/Parkin pathway of mitophagy has been implicated in the pathogenesis of Parkinson’s disease. In prion diseases, a transmissible neurodegenerative disease caused by the misfolded and infectious prion protein (PrP^Sc^), expression of both PINK1 and Parkin are elevated, suggesting that PINK1/Parkin mediated mitophagy may also play a role in prion pathogenesis. Using mice in which expression of either PINK1 (PINK1^KO^) or Parkin (Parkin^KO^) has been ablated, we analyzed the potential role of PINK1 and Parkin in prion pathogenesis. Prion infected PINK1^KO^ and Parkin^KO^ mice succumbed to disease more rapidly (153 and 150 days, respectively) than wild-type control C57Bl/6 mice (161 days). Faster incubation times in PINK1^KO^ and Parkin^KO^ mice did not correlate with altered prion pathology in the brain, altered expression of proteins associated with mitochondrial dynamics, or prion-related changes in mitochondrial respiration. However, the expression level of mitochondrial respiration Complex I, a major site for the formation of reactive oxygen species (ROS), was higher in prion infected PINK1^KO^ and Parkin^KO^ mice when compared to prion infected control mice. Our results demonstrate a protective role for PINK1/Parkin mitophagy during prion disease, likely by helping to minimize ROS formation via Complex I, leading to slower prion disease progression.

## Introduction

Transmissible spongiform encephalopathies, TSE or prion diseases, are a group of rare, fatal, and transmissible neurodegenerative diseases of mammals. They include Creutzfeldt-Jakob disease (CJD) in humans, bovine spongiform encephalopathy (BSE) in cattle, scrapie in sheep, and chronic wasting disease in deer and elk [1]. The causative agent in prion diseases is called a prion. Prions are composed of a misfolded, insoluble, and partially protease resistant form (PrP^Sc^) of the normally soluble and protease sensitive mammalian prion protein (PrP^C^). During prion disease, infectious PrP^Sc^ binds to and changes the conformation of the host PrP^C^ molecule to that of PrP^Sc^, a process of prion replication termed seeded polymerization [2]. Replication and accumulation of PrP^Sc^ within the central nervous system eventually leads to cellular vacuolation and cell loss, culminating in the characteristic spongiform change observed in prion infected brain [3]. Prion diseases thus represent a transmissible neurodegenerative disease. They are always fatal and there are no clinical treatments currently available.

The mechanisms underlying the neurodegeneration that occurs during prion infection are poorly understood and likely multifactorial [3]. One possible contributor to neurodegeneration is the mitochondrion. Mitochondrial dysfunction has been implicated in neurodegenerative diseases such as Alzheimer’s disease (AD) and Parkinson’s disease (PD) [4,5], and there is an increasing body of evidence suggesting that mitochondria can also be impaired in prion disease. A proteomics analysis of prion infected mice suggested that mitochondrial pathways of apoptosis are involved in late-stage prion disease [6]. Changes in mitochondrial structure consistent with mitochondrial damage and impairment have been observed in experimental models of prion infection [7] and can precede the pathological changes that occur early during prion pathogenesis [8]. Altered expression of proteins involved in mitochondrial dynamics, the process of maintaining a healthy pool of mitochondria via cycles of mitochondrial fission and fusion, have been observed in prion infected cells in vitro [9,10], in prion infected rodents [10–13], and in human CJD [10,14]. Signs of dysregulated mitochondrial bioenergetics have also been observed during prion infection, with altered expression levels of multiple proteins within the electron transport chain (ETC) reported in both rodent [8,10,13,15,16] and human [17] prion infection that may contribute to decreased mitochondrial respiration during late stage prion infection [16]. Thus, multiple lines of evidence suggest that mitochondrial dysfunction, both in maintaining a healthy mitochondrial pool and in producing energy for the cell, are significantly impacted during prion infection.

Mitochondrial dysfunction can lead to mitophagy, a crucial process by which damaged mitochondria are degraded by the cell. One of the best understood pathways of mitophagy is regulated by the molecules PTEN induced putative kinase 1 (PINK1) and Parkin (for a recent review see [18]). PINK1 is a serine/threonine protein kinase located on the mitochondrial outer membrane (OMM). In healthy mitochondria, it is translocated into the inner mitochondrial membrane (IMM) and degraded. When mitochondria are damaged, PINK1 is not translocated to the IMM but remains on the OMM where it accumulates, resulting in its autophosphorylation. Phosphorylation of PINK1 triggers the recruitment of the E3 ubiquitin ligase Parkin to the damaged mitochondria where both it and ubiquitin are also phosphorylated by PINK1. Phosphorylation of Parkin activates its ubiquitin ligase activity allowing it to ubiquinate PINK1 and other proteins on the OMM, and the phosphoubiquinated OMM proteins mark the mitochondria for degradation in mitophagosomes [18].

Dysregulation of mitophagy, particularly the pathway mediated by PINK1 and Parkin, has been implicated in multiple neurogenerative diseases [18]. Mutations in Parkin and PINK1 are associated with early onset Parkinson’s disease [19,20] and have been shown in cell culture models to lead to impaired mitophagy [21,22]. Elevated levels of Parkin in AD brain correlate with increased numbers of autophagosomes, suggesting increased levels of mitophagy at earlier stages of disease [23], with Parkin mediated mitophagy decreasing as disease progresses [23]. Similar observations have been made in prion infected cells, mice, and humans. Increased numbers of mitophagosomes and swollen mitochondria have been detected in the mouse prion infected cell line SMB-S15 and in prion infected primary neuronal cultures [9,10], and increased levels of mitophagosomes have been observed in both prion infected mouse and human brain [10]. In addition, increased levels of PINK1 and Parkin have been detected in SMB-S15 cells and in areas of PrP^Sc^ deposition in prion infected mouse brain [9]. These observations are consistent with prion infection inducing increased mitophagy in cells with at least some involvement of the PINK1/Parkin mitophagy pathway.

In order to determine whether or not PINK1 and Parkin contribute to prion pathogenesis, transgenic mice in which either PINK1 or Parkin had been ablated were infected with the rodent adapted mouse prion strain RML and analyzed not only for any effect on disease progression and pathology, but also for the impact on mitochondrial dynamics and bioenergetics. Our results show that, when compared to control wild-type mice expressing both proteins, reduced disease incubation times in the PINK1 and Parkin knockout mice were associated with higher levels of mitochondrial respiration Complex I (CI), a major site of reactive oxygen species (ROS) production. Our results suggest that the PINK1/Parkin mitophagy pathway exerts a protective effect during prion infection, likely slowing disease progression by helping to mitigate mitochondrial ROS production via CI.

## Results

### Decreased prion disease incubation times in RML prion infected PINK1^KO^ and Parkin^KO^ mice

**Fig 1:**
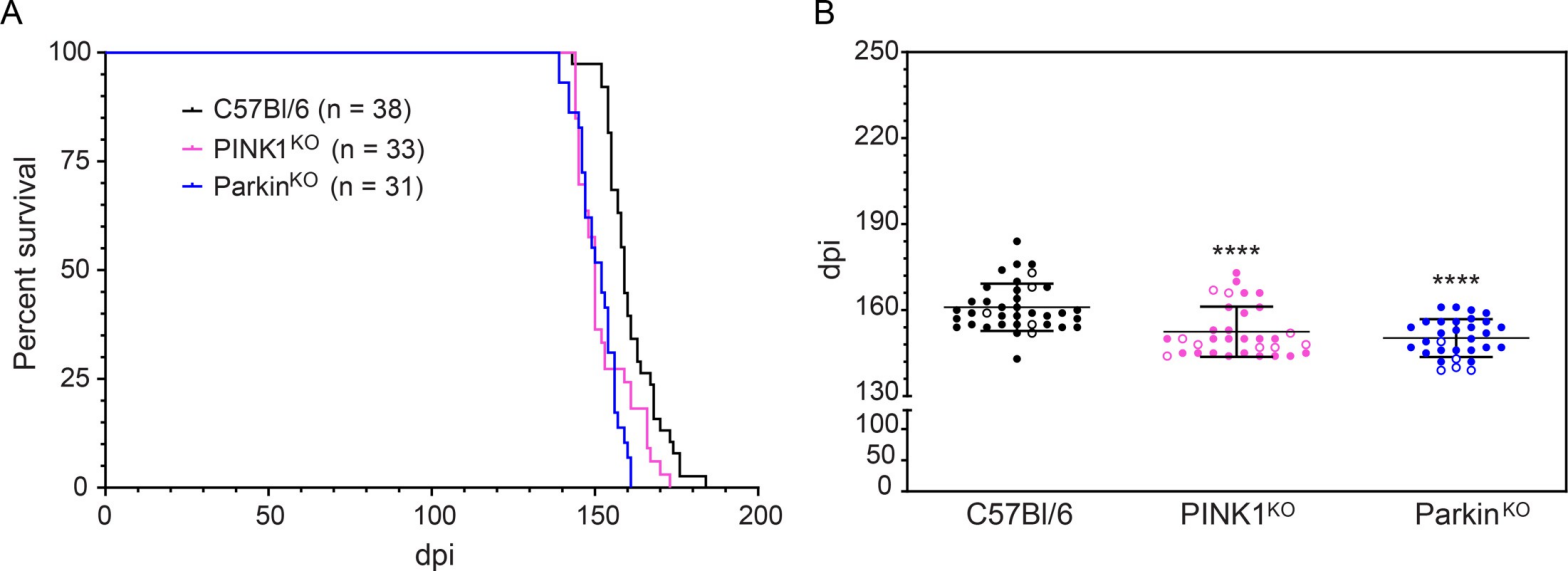
Decreased disease incubation times in RML prion infected PINK1^KO^ and Parkin^KO^ mice. (A) Survival curves for C57Bl/6 (black line), PINK1^KO^ (pink line), and Parkin^KO^ (blue line) mice inoculated intracranially with RML prions. The number of mice inoculated is given in parentheses. Some control mice are included from a previously published study that was done concurrently with the present study [13]. (B) Incubation time scatter plot for the data shown in panel A. Each circle represents one mouse and open circles indicate mice used in the Seahorse XFe96 mitochondrial coupling assay. C57Bl/6 mice used in the Seahorse assay are from a previously published study that was done concurrently with the present study [13]. Mean incubation times plus standard error of the mean (SEM) are indicated and were 161 + 1.3 days for C57Bl/6 mice (n = 38), 152.5 + 1.5 days for PINK1^KO^ mice (n = 33), and 150.3 + 1.2 for the Parkin^KO^ mice (n = 31). Using the Mann-Whitney test, disease incubation times differed significantly for C57Bl/6 versus PINK1^KO^ or Parkin^KO^ mice (****, p value = <0.0001). There was no significant difference in disease incubation times when PINK1^KO^ mice were compared to Parkin^KO^ mice. dpi = days post-inoculation. C57Bl/6 (black circles), PINK1^KO^ (pink circles), and Parkin^KO^ (blue circles).

In order to assess whether or not the PINK1/Parkin pathway of mitophagy influences prion disease pathogenesis, mice ablated for the expression of either PINK1 (PINK1^KO^) [24] or Parkin (Parkin^KO^) [25], as well as wild-type C57Bl/6 control mice, were inoculated intracranially (IC) with RML prions and monitored for disease. RML-infected C67Bl/6 mice had a mean disease incubation time of 161 + 1.3 days post-inoculation (dpi, mean + SEM, Fig 1A). By contrast, RML infected PINK1^KO^ and Parkin^KO^ mice succumbed to disease more quickly, with almost identical mean disease incubation times of 152.5 + 1.5 dpi and 150.3 + 1.2 dpi, respectively (Fig 1A). Disease incubation times in the PINK1^KO^ and Parkin^KO^ mice were significantly different when compared to control C57Bl/6 mice (Fig 1B), indicating that the PINK/Parkin pathway of mitophagy exerts a protective effect during prion disease.

**Fig 2:**
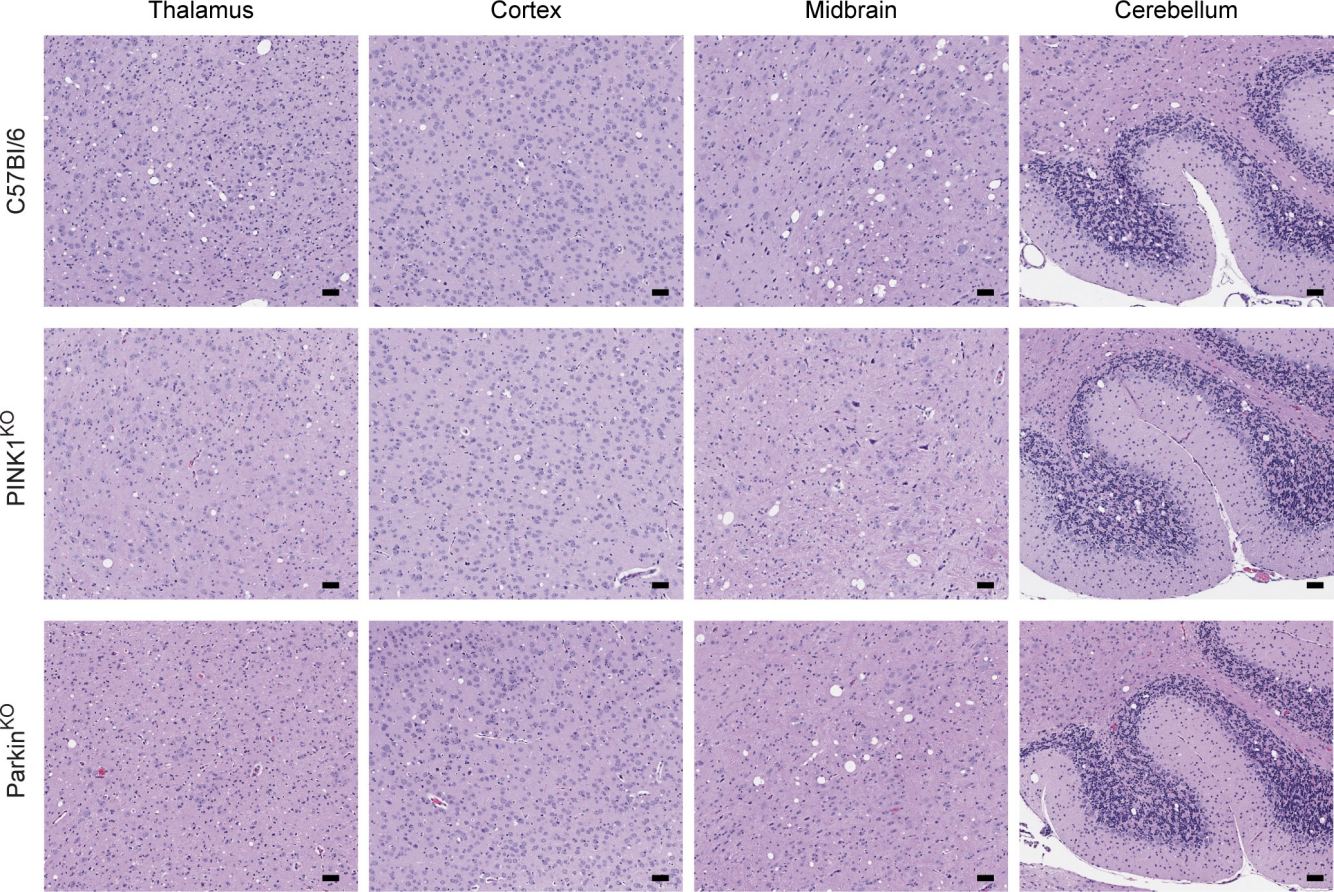
Similar distribution of spongiform change in the brains of RML prion infected C57Bl/6, PINK1^KO^, and Parkin^KO^ mice. H&E staining for RML prion infected C57Bl/6 (155 dpi), PINK1^KO^ (144 dpi), and Parkin^KO^ (144 dpi) mice. Spongiform change in representative sections from thalamus, cortex, midbrain, and cerebellum is shown. For all panels, scale bar = 50μm.

**Fig 3:**
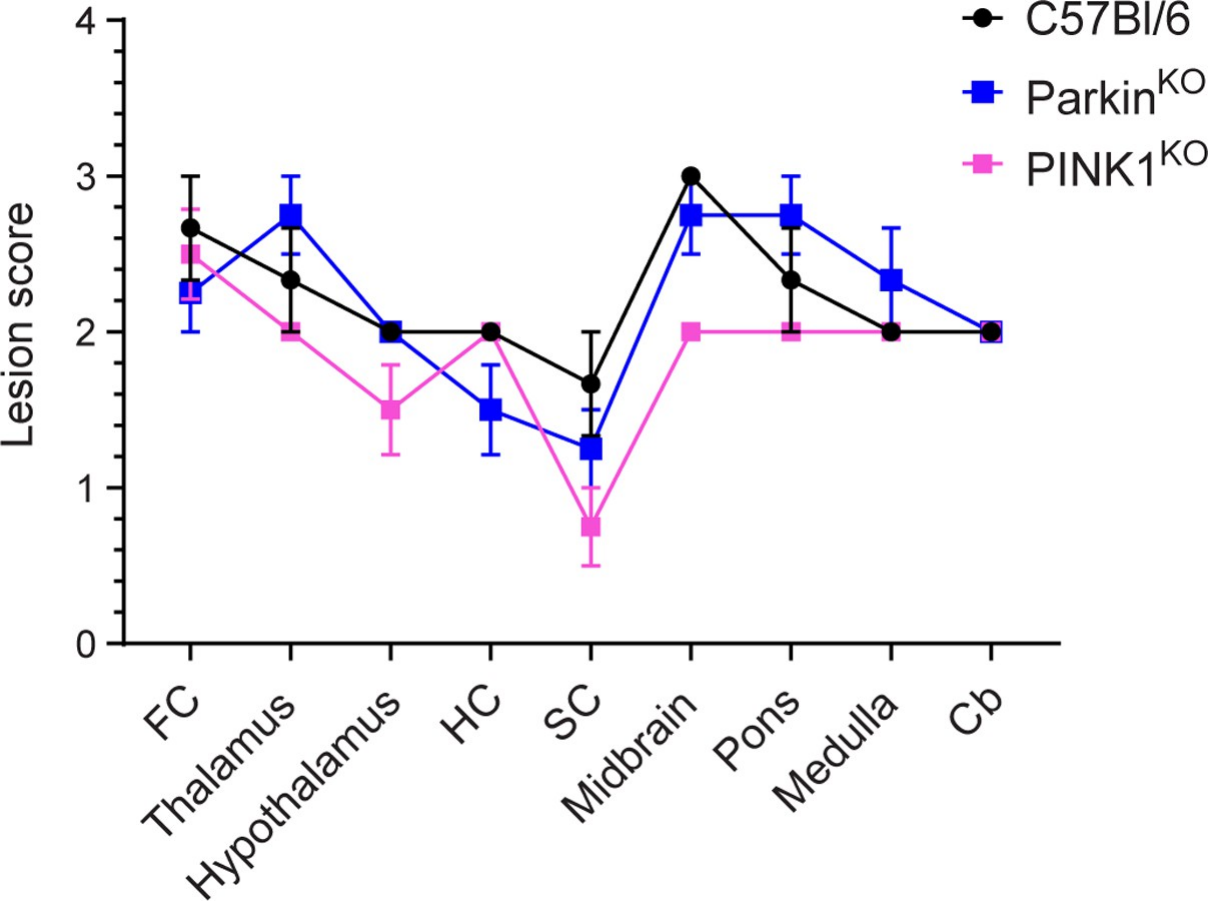
Similar lesion profiles for RML prion infected C57Bl/6, PINK1^KO^, and Parkin^KO^ mice. Brain lesion profile of clinically positive RML infected C57Bl/6 (n = 3, black line and filled circles), PINK1^KO^ (n = 4, pink line and filled squares), and Parkin^KO^ (n = 4, blue line and filled squares) mice. Mean ± S.E.M. is shown. FC = frontal cortex; HC = hippocampus; SC = superior colliculus; Cb = cerebellum. C57Bl/6 control mice are from a previously published study that was done concurrently with the present study [13].

The protective effect of the PINK1/Parkin pathway during prion infection may be related to the role of mitophagy in protecting against neuronal loss [26]. It was thus possible that the level of spongiform change in prion infected mice would be greater in the absence of PINK1 and Parkin than in control mice expressing both proteins. We therefore examined the distribution and intensity of spongiform change throughout the brains of infected mice. In negative control mice inoculated with normal brain homogenate (NBH), no spongiform change was observed, indicating that lack of either PINK1 or Parkin did not lead to significant spongiform pathology in the brain (S1 Fig). In RML infected mice, spongiform change was widespread throughout the brain in all three mouse strains, with no difference in distribution between control C57Bl/6, PINK1^KO^, and Parkin^KO^ mice (Fig 2). While there appeared to be slightly less spongiosis in the midbrain of PINK1^KO^ mice (Fig 3), overall there were similar levels of spongiform change across multiple brain regions for all three mouse lines (Fig 3). Thus, loss of the PINK1/Parkin pathway of mitophagy did not appear to lead to significantly increased vacuolation or cell loss in the brains of prion infected mice.

**Fig 4:**
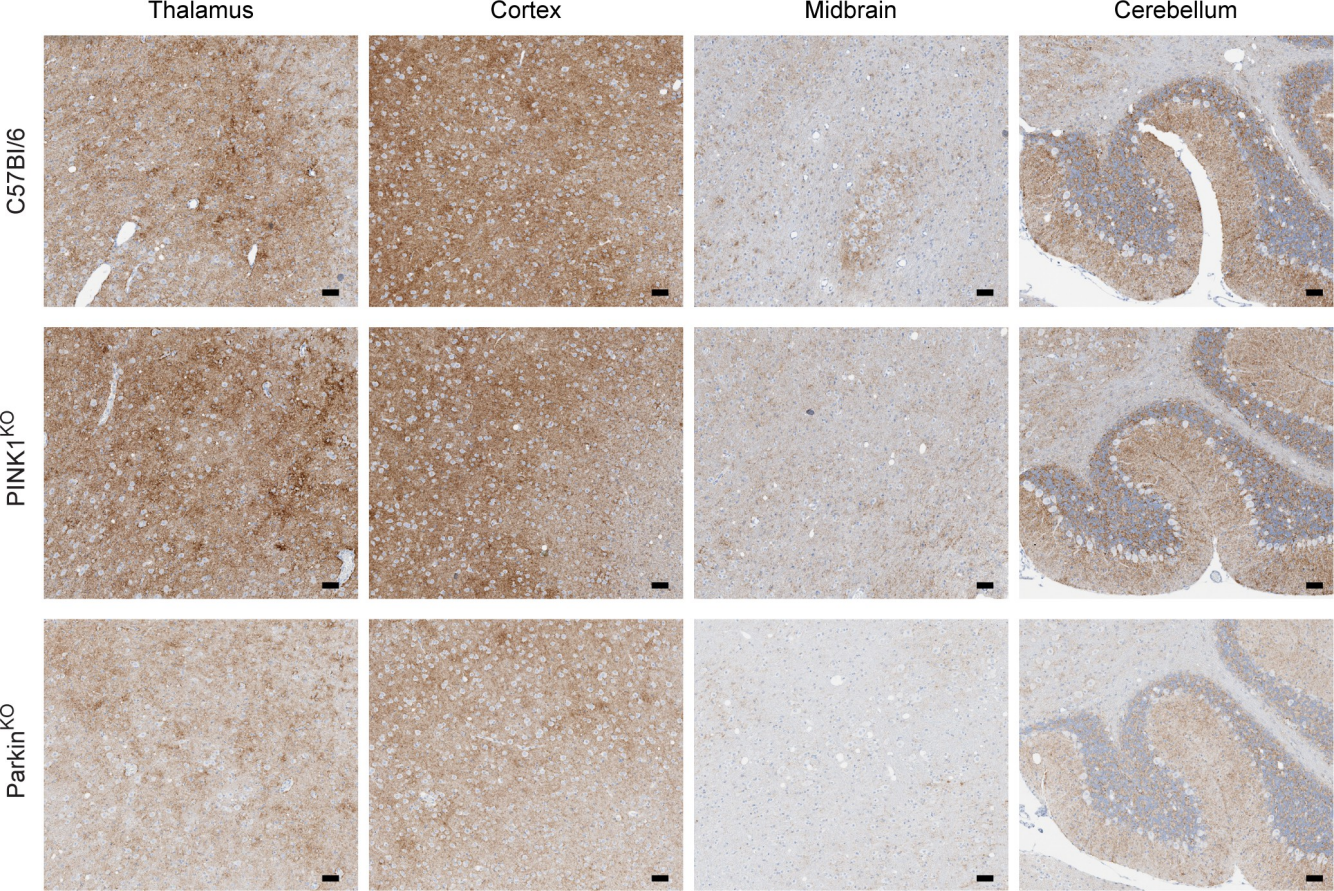
PrP^Sc^ deposition and distribution in the brain is similar at the clinical stage of disease in C57Bl/6, PINK1^KO^, and Parkin^KO^ mice inoculated with RML prions. PrP^Sc^ staining for RML prion infected C57Bl/6 (155 dpi), PINK1^KO^ (144 dpi), and Parkin^KO^ (144 dpi) mice. PrP^Sc^ deposition in representative sections from thalamus, cortex, midbrain, and cerebellum is shown. The sections are from the same mice used in Fig 2. PrP^Sc^ was detected using the anti-PrP rabbit monoclonal antibody EP1802Y as described in the Materials and Methods. For all panels, scale bar = 50μm.

**Fig 5:**
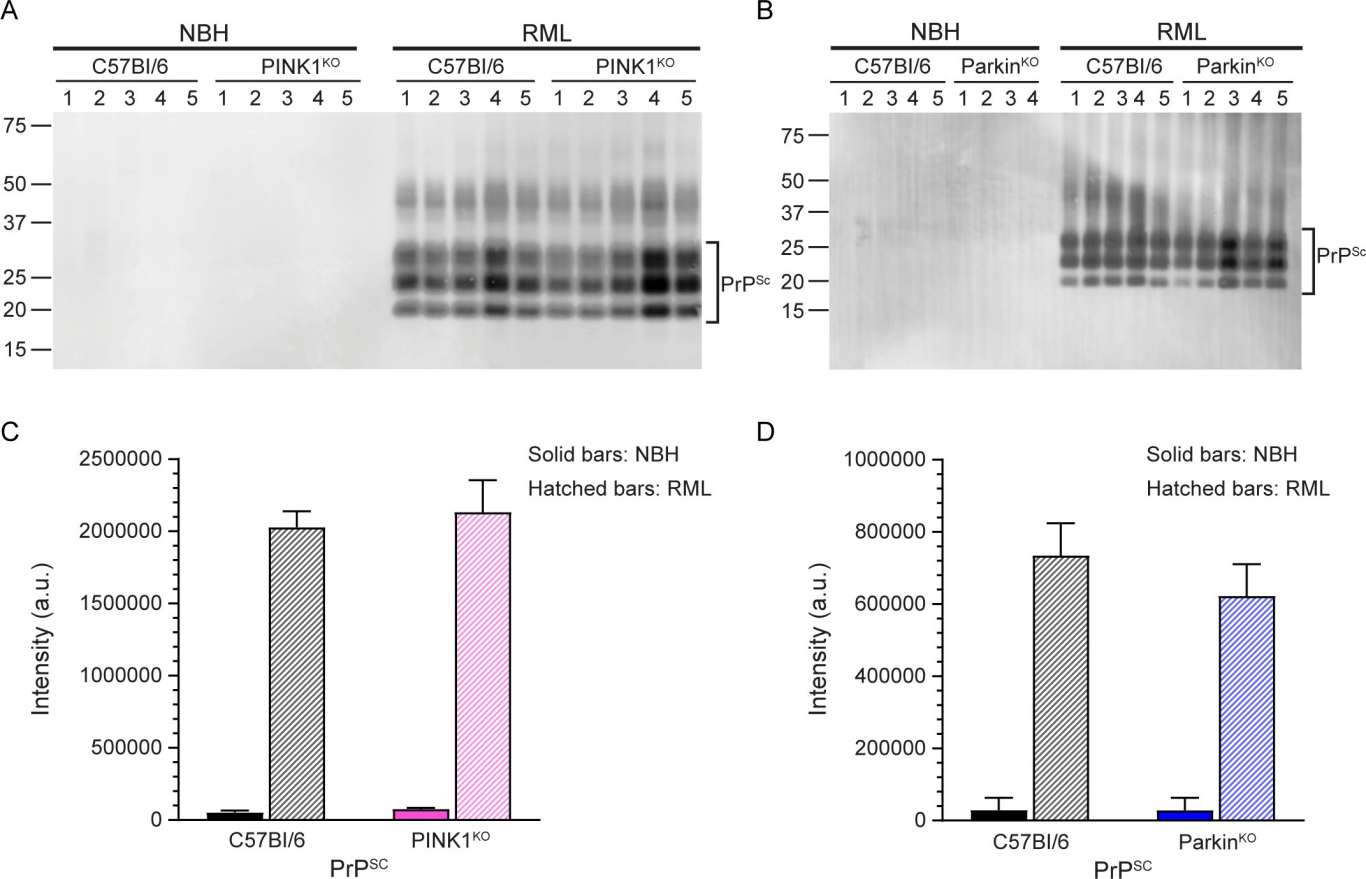
RML prion infected C57Bl/6, PINK1^KO^, and Parkin^KO^ mice accumulate similar levels of PrP^Sc^ in the brain. Western blot analysis of PrP^Sc^ in the brains of mice inoculated with normal brain homogenate (NBH) or RML prion infected brain homogenate (RML). Samples from the same control C57Bl/6 mice were loaded on each blot to facilitate direct quantitation of the amount of PrP^Sc^ in wild-type versus transgenic knockout mice. All samples were PK treated as described in the Materials and Methods. (A) C57Bl/6 (n = 5) and PINK1^KO^ (n = 5) mice. (B) C57Bl/6 (n = 5) and Parkin^KO^ (NBH n = 4, RML n = 5) mice. For panels A and B, the sample number is given above each lane and unlabeled lanes are empty. Irrelevant lanes have been excised but can be seen in the Fig 5 raw image files in S1 Raw images. Blots were developed using the anti-PrP mouse monoclonal antibody 6D11. Molecular mass markers in kilodaltons are shown on the left. (C) Quantitation of the amount of PrP^Sc^ in C57Bl/6 (black bars) or PINK1^KO^ (pink bars) mice inoculated with NBH (solid bars) or RML prions (hatched bars). (D) Quantitation of the amount of PrP^Sc^ in C57Bl/6 (black bars) or Parkin^KO^ (blue bars) mice inoculated with NBH (solid bars) or RML prions (hatched bars). Quantitation data in panels C and D are based on the analysis of bands from duplicate gels that were run independently and are given as mean + SEM. There was no significant difference in PrP^Sc^ levels in wild-type versus transgenic knockout mice.

We also analyzed the distribution and pattern of PrP^Sc^ staining to see if lack of PINK1 or Parkin affected PrP^Sc^ deposition. While control brains inoculated with NBH were negative for PrP^Sc^ (S2 Fig), a diffuse, punctate pattern of PrP^Sc^ deposition consistent with infection by RML prions was observed in multiple brain regions of prion infected mice (Fig 4). While there appeared to be slightly less PrP^Sc^ in the Parkin^KO^ brain (Fig 4), this difference was not significant by western blot analysis (Fig 5A and 5B) which confirmed that overall PrP^Sc^ levels in prion infected C57Bl/6, PINK1^KO^, and Parkin^KO^ mice were similar (Fig 5C and 5D). The lack of either PINK1 or Parkin therefore did not appear to have any significant influence on the distribution or amount of PrP^Sc^ deposition.

**Fig 6:**
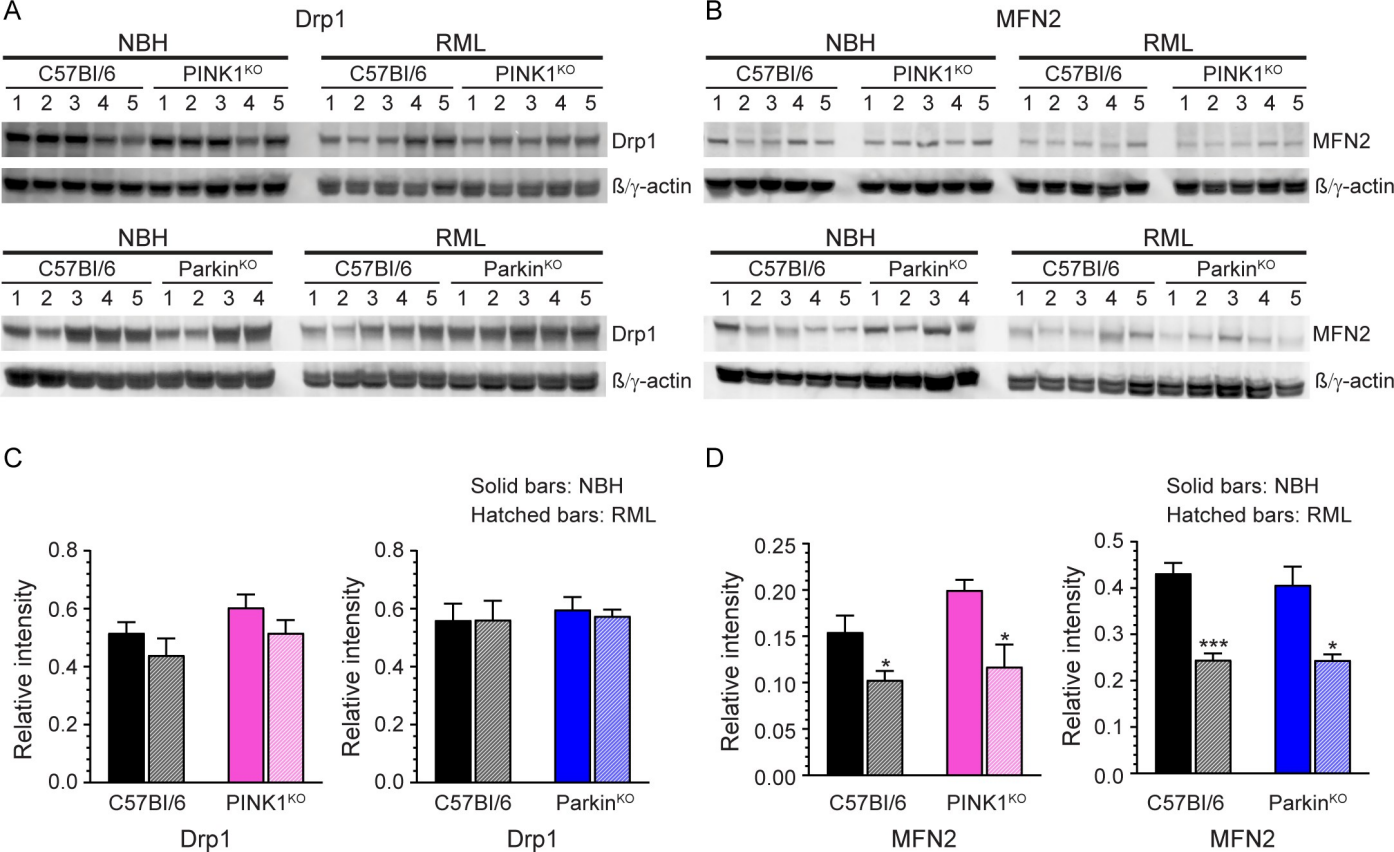
Expression level of proteins involved in mitochondrial dynamics in prion infected C57Bl/6, PINK1^KO^, and Parkin^KO^ mice. (A) Representative immunoblots for Drp1 and mouse β/γ actin from C57Bl/6 and PINK1^KO^ mice (upper panel) or C57Bl/6 and Parkin^KO^ mice (lower panel) inoculated IC with normal brain homogenate (NBH) or RML prion infected brain homogenate (RML). Sample number is shown above each lane. Data are from the same gel with intervening gel space between the bands of interest spliced out. (B) Representative immunoblots for MFN2 and mouse β/γ actin from C57Bl/6 and PINK1^KO^ mice (upper panel) or C57Bl/6 and Parkin1^KO^ mice (lower panel) inoculated IC with NBH or RML prions. Data are from the same gel with intervening gel space between the bands of interest spliced out. For panels A and B, samples from the same control C57Bl/6 mice were loaded on each blot to facilitate direct quantitation of the amount of Drp1 or MFN2 in wild-type versus transgenic knockout mice. The antibodies used for both panels are described in the Materials and Methods and the full blots can be seen in the Fig 6A and 6B in S1 Raw images. (C) Quantitation of the amount of Drp1 in C57Bl/6 (black bars), PINK1^KO^ (pink bars), and Parkin^KO^ (blue bars) mice inoculated with NBH (solid bars) or RML prions (hatched bars). (D) Quantitation of the amount of MFN2 in C57Bl/6 (black bars), PINK1^KO^ (pink bars), and Parkin^KO^ (blue bars) mice inoculated with NBH (solid bars) or RML prions (hatched bars). Quantitation data in panels C and D are based on the analysis of bands from duplicate gels that were run independently and are given as mean + SEM. Data were normalized to mouse β/γ actin (Relative intensity) and were calculated from n = 4–5 animals for each condition. Significance was calculated by comparing the NBH and RML samples for each condition using the unpaired Student’s t-test with Welch’s correction. * p value = 0.02–0.04; *** p value = 0.0003.

Mitochondrial dynamics is the process for maintaining healthy mitochondria via mitochondrial fusion and fission. Mitochondrial dynamics are disrupted during prion disease, where expression levels of a key protein involved in mitochondrial fission, dynamin-related protein 1 (Drp1) [10–13], and a key protein involved in mitochondrial fusion, mitofusion 2 (MFN2) [9,11,13], are altered. Since the PINK1/Parkin pathway can regulate mitochondrial dynamics by promoting mitochondrial fission via the expression level of PINK1 [27], we hypothesized that the lack of this pathway would exacerbate the disruption to mitochondrial dynamics observed during prion disease. We therefore analyzed the expression levels of both Drp1 (Fig 6A) and MFN2 (Fig 6B) in brain homogenates from infected and uninfected PINK1^KO^ and Parkin^KO^ mice and compared it to prion infected C57Bl/6 mice. Contrary to what has been previously reported [10–12], there was no significant change in the expression level of the mitochondrial fission protein Drp1 in prion infected wild-type mice when compared to uninfected mice (Fig 6C). Drp1 expression was also unchanged in prion infected PINK1^KO^ and Parkin^KO^ mice, with no difference observed between the two knockout mouse lines and wild-type C57Bl/6 mice (Fig 6C). By contrast, and consistent with other studies [11,13], MFN2 levels were decreased in prion infected mice from all three mouse strains, with no significant difference between PINK1^KO^, Parkin^KO^, and wild-type mice (Fig 6D). Overall, our data suggest that lack of either PINK1 or Parkin does not significantly alter the changes in key proteins involved in mitochondrial dynamics that occur during prion infection.

We have recently shown that mitochondrial respiration, the process of oxidative phosphorylation whereby mitochondria consume oxygen to produce ATP, can be impaired during prion infection [16]. We have also found that faster prion disease incubation times correlated with increased mitochondrial respiration in prion infected mice lacking the molecule SARM1 [13], a protein that may also be involved in the PINK1/Parkin pathway of mitophagy [28]. We therefore asked whether or not mitochondrial respiration was similarly increased in prion infected PINK1^KO^ and Parkin^KO^ mice. Mitochondria were isolated from the brains of clinically positive C57Bl/6, PINK1^KO^, and Parkin^KO^ mice inoculated with RML prions (open circles in Fig 1B) or clinically normal mice inoculated with NBH. Oxidative phosphorylation in response to succinate, the substrate for the mitochondrial ETC complex II (CII), was then analyzed using a Seahorse XF Analyzer as previously described [13].

**Fig 7:**
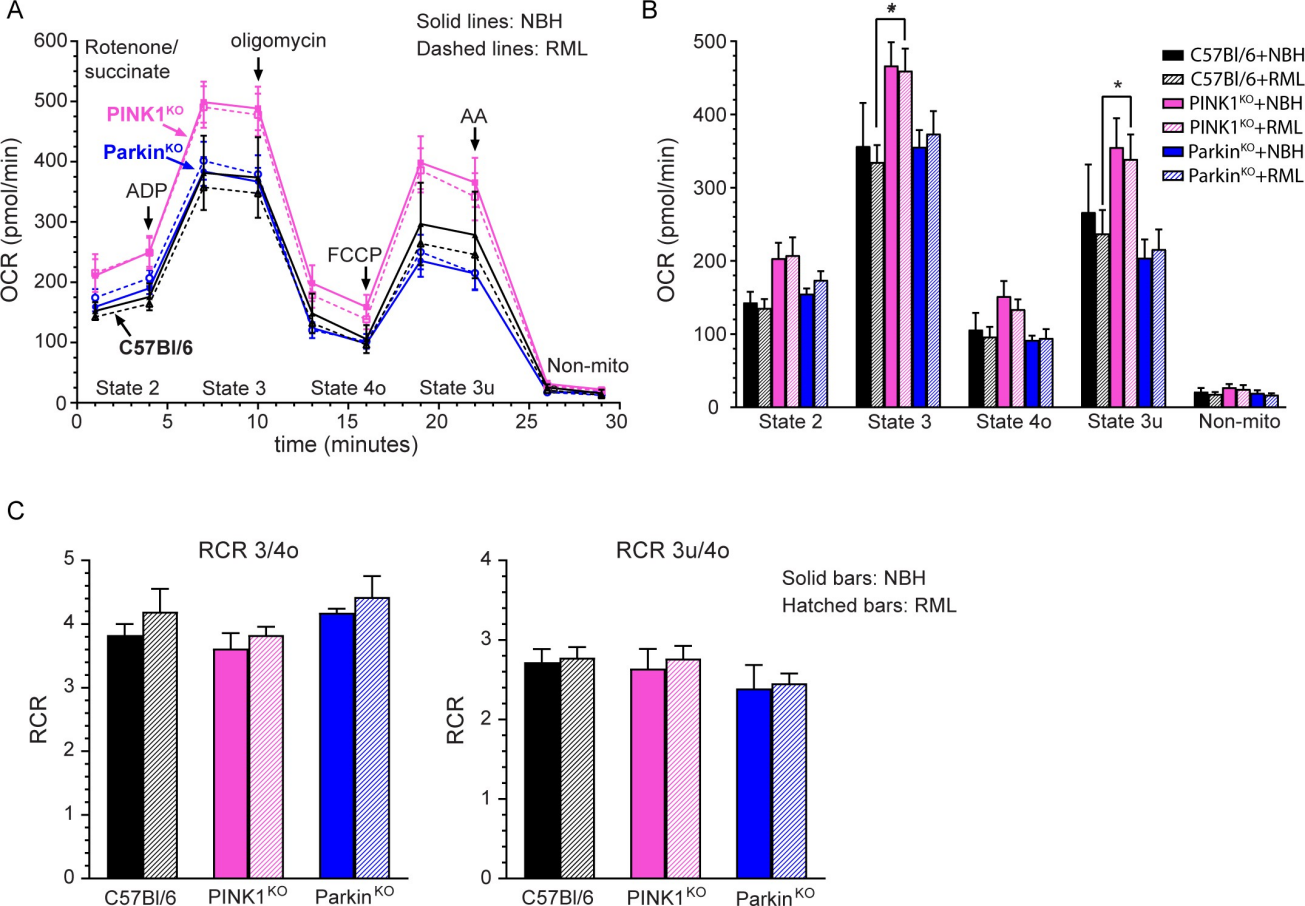
Lack of PINK1 and Parkin does not significantly impact mitochondrial respiration during prion infection. (A) Mitochondrial coupling assay measuring mitochondrial respiration in response to succinate for C57Bl/6 (n = 5, triangles and black lines), PINK1^KO^ (n = 8 for NBH, n = 9 for RML, squares and pink lines), and Parkin^KO^ (n = 5, circles and blue lines) mice inoculated with normal brain homogenate (NBH, closed symbols and solid lines) or clinically positive mice inoculated with RML prion infected brain homogenate (RML, open symbols and dashed lines). The reagents added during the 30 min assay as well as the states quantified are indicated. AA = antimycin A; Non-mito = non mitochondrial respiration. (B) OCRs and (C) RCRs for the data in Panel A. Data were calculated as described in the Materials and Methods. Black bars = C57BL/6, pink bars = PINK1^KO^, blue bars = Parkin^KO^. Solid bars represent mice inoculated with NBH while hatched bars represent mice infected with RML prions. Mean + SEM is shown. Statistical significance was calculated using a 1-way ANOVA with Dunnett’s post-test with C57Bl/6 samples set as the control. * p value = 0.019. C57Bl/6 mice used in the Seahorse assay are from a previously published study that was done concurrently with the present study [13].

As shown in Fig 7A, the oxygen consumption rates (OCRs) of mitochondria isolated from C57Bl/6 and Parkin^KO^ mice inoculated with either RML prions or NBH were similar, with no difference in the mitochondrial OCRs between prion infected and control mice inoculated with NBH. However, mitochondria isolated from PINK1^KO^ mice inoculated with either RML prions or NBH tended to have higher OCRs than mitochondria from either the C57Bl/6 or Parkin^KO^ inoculated mice (Fig 7A). While basal respiration (State 2), proton leak (State 4o), oxidative capacity (State 3u), and non-mitochondrial respiration were similar for mitochondria isolated from all of the mice inoculated (Fig 7B), oxidative phosphorylation (State 3) was significantly higher for mitochondria isolated from prion infected PINK1^KO^ mice when compared to the wild-type C57Bl/6 controls (Fig 7B). However, it was not higher than the State 3 OCR of mitochondria from PINK1^KO^ mice inoculated with NBH (Fig 7B), suggesting that the increase in mitochondrial OCR was not related to prion infection. Finally, the respiratory control ratios (RCRs) of state 3/state 4o and state 3u/state 4o, which indicate how tightly mitochondrial oxygen consumption and ADP phosphorylation are coupled, were similar between mitochondria from all three mouse strains (Fig 7C) and consistent with previously reported RCRs for healthy brain mitochondria [29]. Overall, there appeared to be little change in mitochondrial respiration in response to RML prion infection in any of the mouse strains tested.

Age related changes in mitochondrial function have been reported for the PINK1^KO^ mice [30]. It was therefore possible that the higher rate of oxidative phosphorylation and oxidative capacity observed in the PINK1^KO^ mice, while unrelated to prion infection, was an age-related change that occurred over the course of the experiment. Analysis of mitochondrial respiration in response to succinate in mitochondria isolated from the brains of C57Bl/6, PINK1^KO^, and Parkin^KO^ mice showed no difference between the OCRs in normal, uninoculated, younger mice versus uninoculated but aged mice (S3A Fig). However, the OCRs of the different states tended to be higher in PINK1^KO^ and Parkin^KO^ mice when compared to the C57Bl/6 controls, with some differences reaching a modest level of significance in the aged mice (S3B Fig). Oxidative phosphorylation and oxygen consumption were tightly coupled for all groups analyzed, with only a small, albeit significant, difference between aged PINK1^KO^ and control C57Bl/6 mice (S3C Fig). Overall, the data suggest that age was unlikely to be a major contributing factor to the higher state 3 OCR observed in IC inoculated PINK1^KO^ mice.

### Altered expression of mitochondrial respiratory complex proteins in RML infected wild-type and knockout mice

**Fig 8:**
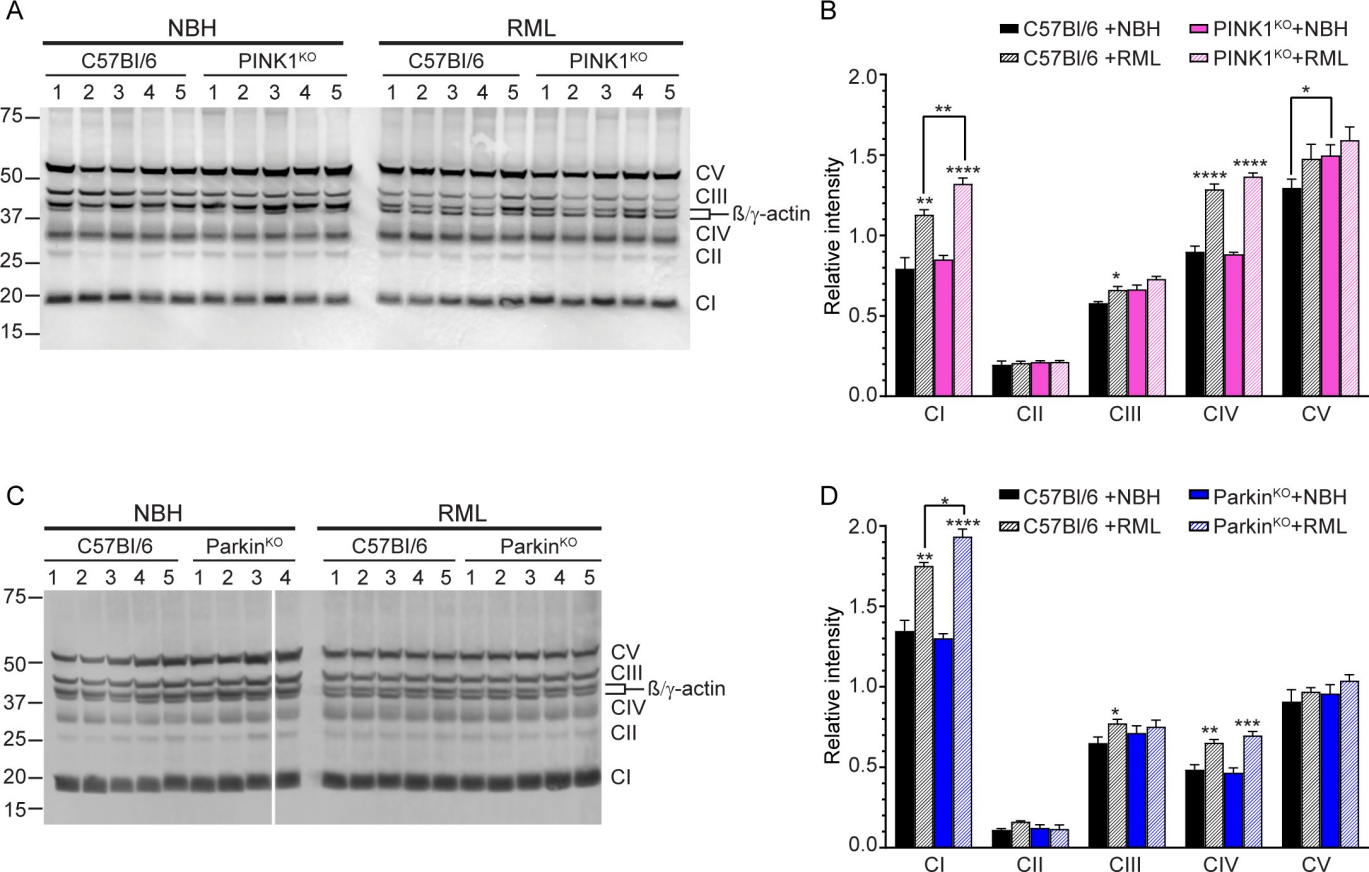
Increased expression of mitochondrial complex proteins in prion infected mice. (A) Immunoblot of proteins in mitochondrial complexes CI-CV in C57Bl/6 and PINK1^KO^ mice inoculated with either normal brain homogenate (NBH) or RML prion infected brain homogenate (RML). The immunoblot was developed using the Total OXPHOS Rodent WB Antibody Cocktail and an antibody to mouse β/γ-actin. Complex I-V proteins indicated on the right side of the panel are: CI, NADH:ubiquinone oxidoreductase subunit B8; CII, succinate dehydrogenase [ubiquinone] iron-sulfur subunit B; CIII, cytochrome b-c1 complex subunit 2, CIV, cytochrome c oxidase subunit I; CV, ATP synthase subunit alpha. β/γ-actin is indicated on the right side of the panel and molecular mass markers in kDa are indicated on the left. Irrelevant lanes have been excised but can be seen in the Fig 8A raw image file in S1 Raw images. (B) Quantitation (mean + SEM) of the expression level of mitochondrial complexes CI-CV in C57Bl/6 (black bars) and PINK1^KO^ (pink bars) mice inoculated with NBH (solid bars) or RML prions (hatched bars). (C) Immunoblot of proteins in mitochondrial complexes CI-CV in C57Bl/6 and Parkin^KO^ mice inoculated with NBH or RML prions. Immunoblot development and labeling are as in Panel A. The thin white line indicates an irrelevant excised lane that can be seen in the Fig 8C raw image file in S1 Raw images. (D) Quantitation (mean + SEM) of the expression level of mitochondrial complexes CI-CV in C57Bl/6 (black bars) and Parkin^KO^ (blue bars) mice inoculated with NBH (solid bars) or RML prions (hatched bars). Quantitation data in panels B and D are based on the analysis of bands from duplicate gels that were run independently and are given as mean + SEM. Data were normalized to mouse β/γ actin (Relative intensity) and were calculated from n = 4–5 animals for each condition. Significance was calculated using the unpaired Student’s t-test with Welch’s correction between RML and NBH inoculated mice unless otherwise indicated by the brackets. * p value = 0.01–0.03; ** p value = 0.003; *** p value = 0.0001–0.0002; **** p value = <0.0001.

Changes in expression level of the proteins that make up the five complexes (CI-CV) responsible for mitochondrial respiration have been reported in prion disease in humans [17] and rodents [8,13,16]. We therefore analyzed a key protein in each of the complexes to determine whether there were any changes in expression level that could help explain both the shortened disease incubation times in prion infected PINK1^KO^ and Parkin^KO^ mice and the higher OCR observed in IC inoculated PINK1^KO^ mice. The proteins analyzed by western blot were NADH:ubiquinone oxidoreductase subunit B8 in CI, succinate dehydrogenase [ubiquinone] iron-sulfur subunit B in CII, cytochrome b-c1 complex subunit 2 in CIII, cytochrome c oxidase subunit I in CIV, and ATP synthase subunit alpha of CV (Fig 8A and 8C).

In all 3 mouse lines, prion infection led to significant increases in CI and CIV when compared to NBH inoculated controls (Fig 8B and 8D). However, the expression level of CI in C57Bl/6 was significantly lower than that observed in either the PINK1^KO^ or Parkin^KO^ mice (Fig 8B and 8D), suggesting that prion infection in the absence of PINK1 and Parkin has a greater effect on CI levels. We also observed a small but significant increase in CIII in RML inoculated C57Bl/6 mice (Fig 8B and 8D) and a slight but significant increase in CV in the NBH inoculated PINK1^KO^ mice that was unrelated to prion infection (Fig 8B). Overall, the data suggest that CI levels are differentially affected in a significant way following prion infection of either the PINK1^KO^ or Parkin^KO^ mice.

## Discussion

The PINK1/Parkin pathway of mitophagy has been implicated in multiple neurodegenerative diseases including prion diseases [18]. However, direct evidence for a role of PINK1/Parkin mediated mitophagy in modulating neurodegenerative disease pathogenesis in animal models is limited [18]. For example, although regulation of dopamine appears to be affected [30,31], there is no loss of dopaminergic neurons in PINK1^KO^ [24] and Parkin^KO^ mice [25], and they do not develop any PD-like pathology [24,25,30–32]. We have now shown that lack of expression of either PINK1 or Parkin leads to a more rapid progression of prion disease, with similar prion disease incubation times in mice ablated for either protein (Fig 1). Not only prion disease incubation times but also prion pathology, PrP^Sc^ deposition, expression of proteins associated with mitochondrial dynamics, and prion infection related increases in mitochondrial ETC proteins were all indistinguishable between the two strains. This strongly suggests that it is the PINK1/Parkin pathway of mitophagy, where both proteins have key roles, that is exerting a protective effect during the host response to prion infection. To our knowledge, our data are the first to demonstrate that expression of PINK1 and Parkin helps to slow the progression of a neurodegenerative disease *in vivo*. Furthermore, since prion disease in mice largely recapitulates prion disease in humans with regard to clinical signs, pathology, and PrP^Sc^ properties [33], it is reasonable to assume that PINK1 and Parkin may exert a similar protective effect on human forms of prion disease such as CJD.

Changes in the relative expression of proteins associated with mitophagy have been observed in prion disease and are indicative of impaired mitochondrial dynamics and mitophagy [9–13,34]. Elevated levels of both PINK1 and Parkin have been observed in prion infected cells *in vitro* and in prion infected brain *in vivo* [9]. This has led to the hypothesis that excessive mitophagy may be occurring during prion disease, leading to impaired mitochondrial energetics and increased ROS, ultimately culminating in the neuronal apoptosis and spongiform change that are characteristic of prion infection [10]. A similar mechanism has been proposed for AD. Increased levels of mitochondria-associated Parkin have been detected in transgenic models of AD as well as in AD brain early during disease progression [23], while other studies have shown that excessive recruitment of Parkin can lead to dysregulation of mitophagy, synaptic degeneration, and neuronal death [35].

If excessive PINK1/Parkin mediated mitophagy is an important contributor to mitochondrial dysfunction and ROS leading to neuronal apoptosis and spongiform change during prion infection, then lack of PINK1 or Parkin would presumably slow the neurodegenerative process and lead to increased incubation times. However, disease incubation times were longer in wild-type mice that express either PINK1 or Parkin when compared to mice where either PINK1 or Parkin were ablated (Fig 1). This strongly suggests that PINK1/Parkin mediated mitophagy actually helps to slow disease progression and is consistent with the observation that mutant forms of PINK1 and Parkin that impair mitophagy are associated with early onset PD in humans [19–22]. Also arguing against excessive PINK1/Parkin mitophagy negatively impacting prion disease is the fact that we did not observe any differences between prion infected C57Bl/6 mice, PINK1^KO^, and Parkin^KO^ mice with regard to the overall expression of two proteins involved in mitochondrial dynamics, MFN2 and Drp1. In all 3 lines, expression of the mitochondrial fusion protein MFN2 was significantly decreased compared to NBH inoculated controls while expression of the mitochondrial fission protein Drp1 was not significantly changed (Fig 6). This is consistent with previous work from our laboratory [13] and others [9], and suggests a similar shift in mitochondrial dynamics during prion infection from mitochondrial fusion to mitochondrial fission and mitophagy in all three mouse lines. Finally, lack of either PINK1 or Parkin did not alter the pattern and intensity of spongiform change (Figs 2 and 3) or the amount of PrP^Sc^ in the brains of prion infected mice at the end stage of disease (Fig 5). Taken together, our data support a protective role for mitophagy in prion disease and are inconsistent with the hypothesis that the elevated levels of PINK1 and Parkin observed during prion disease [9] are indicative of excessive mitophagy that exerts a negative effect on prion disease outcome.

As noted above, we observed very little difference between PINK1^KO^, Parkin^KO^ and wild-type mice with regard to spongiform change as well as two major proteins involved in mitochondrial dynamics. It’s possible that, in the absence of PINK1 or Parkin, mitophagy levels were differentially impacted only in certain brain regions. It’s also possible that other proteins associated with mitophagy may be at least partially compensating for the loss of PINK1 or Parkin [9]. For example, in the absence of Parkin the E3 mitochondrial ubiquitin ligase protein MUL1 can stabilize PINK1 on the OMM and trigger ubiquitin-mediated mitophagy [36], while phosphoubiquination by PINK1 alone is sufficient to recruit other autophagy receptors to the mitochondria to induce mitophagy [37]. The Nip3-like protein X (Nix or BNIP3L), a mitochondrial OMM autophagy receptor that helps to mediate mitophagy [38], has been shown to compensate for the loss of functional Parkin or PINK1 in genetic Parkinson’s disease [39]. Thus, there is evidence that other proteins can act in the absence of either PINK1 or Parkin to induce mitophagy. Since our data suggest that PINK1/Parkin mediated mitophagy is protective during prion infection, it’s possible that prion disease would progress even faster if mice were ablated for MUL1 or Nix, in addition to PINK1 or Parkin.

We did not observe any prion-specific effect on succinate driven mitochondrial respiration in PINK1^KO^ or Parkin^KO^ mice, with both NBH and RML prion inoculated mice showing similar OCR rates for basal respiration (State 2), phosphorylating respiration (State 3), and oxidative capacity (State 3u), as well as similar levels of proton leak (Sate 4o) (Fig 7). OCRs for State 3 were significantly higher in RML prion inoculated PINK1^KO^ mice when compared to RML inoculated wild-type mice, but they were also higher in NBH inoculated PINK1^KO^ mice. This suggests that, regardless of prion infection, mitochondria in PINK1^KO^ mice may have a higher overall rate of oxidative phosphorylation. Consistent with this, uninoculated and aged PINK1^KO^ and Parkin^KO^ also trended towards higher OCRs for State 3 when compared to uninoculated and aged wild-type mice (S3 Fig), although only the State 3 OCR for the aged Parkin^KO^ mice was modestly significant.

The higher State 3 OCR in RML inoculated PINK1^KO^ mice suggests that regulation of mitochondrial bioenergetics is perturbed. Consistent with this, we observed higher levels of ETC complex CI in the PINK1^KO^ mice when compared to both NBH inoculated PINK1^KO^ mice and prion infected wild-type mice (Fig 8). However, we also observed increases in CI above wild-type levels in prion infected Parkin^KO^ mice (Fig 8), even though the State 3 OCR in these mice was similar to that of wild-type mice (Fig 7). Thus, increases in the level of CI above those of wild-type mice did not appear to correlate well with increased oxidative phosphorylation. Interestingly, all three prion infected mouse lines showed increases in complexes CI and CIV when compared to NBH inoculated mice (Fig 8). CI is a major source of ROS production in mitochondria via consumption of NADH [40], with the ratio of NADH to NAD^+^ significantly influencing the amount of ROS produced [40,41]. Increased levels of CI during prion infection could impact consumption of NADH and thus alter the NADH/NAD^+^ ratio, increasing the amount of ROS generated by CI. This effect could be exacerbated by the higher levels of CI in both the PINK1^KO^ and Parkin^KO^ mice, with the accompanying increase in CIV possibly related to its function as the rate limiting step in oxidative phosphorylation [42]. Consistent with this idea, studies in both prion infected mice ablated for the NADase SARM1 [13] and in an *in vitro* cell model of prion neurotoxicity [43] are also supportive of the hypothesis that regulation of the NADH/NAD^+^ ratio helps to mitigate the damage caused by oxidative stress and slow prion disease progression [13].

We chose to use mitochondria isolated from whole brain because of the widespread effects of prion infection in the brain (Figs 2–4). However, earlier studies using mitochondria isolated from specific brain regions of PD patients and transgenic mice have yielded some contrasting data. Decreased, not increased, CI levels were observed in mitochondria isolated from the substantia nigra of a PD patient [44], in a cell model of PD mitochondrial function [45], and in the ventral midbrain of Parkin^KO^ mice [32]. State 3 respiration was also decreased, not increased, in mitochondria isolated from the striatum of both PINK1^KO^ and Parkin^KO^ mice [30,32]. The use of mitochondria from whole brain versus specific brain regions likely accounts for these differences since the use of whole brain could obscure any brain region specific effects due to lack of PINK1 or Parkin. Importantly, our hypothesis that PINK1 and Parkin may help to mitigate the damage caused by oxidative stress during prion infection is supported by previous work in both mouse lines. PINK1^KO^ mitochondria are more susceptible to stress induced by hydrogen peroxide [30] while Parkin^KO^ mice are more susceptible to ROS mediated damage [32]. Overall, our data are consistent with the PINK1/Parkin pathway of mitophagy helping to slow the progression of a neurodegenerative disease *in vivo* by maintaining healthy, functional mitochondria and limiting the damage induced by ROS generated by dysfunctional mitochondria.

## Materials and methods

### Infection of mice

Animal protocols (2015-004E, 2018-024E) were reviewed and approved by the Rocky Mountain Laboratories Animal Care and Use Committee. This study was carried out in strict accordance with the recommendations in the *Guide for the Care and Use of Laboratory Animals* of the National Institutes of Health. Animals in distress or showing symptoms of clinical prion disease were euthanized by isofluorane overdose followed by cervical dislocation.

Breeding pairs of mice ablated for the expression of PINK1 (B6.129S4-*Pink1*^*tm1Shn*^/J, hereafter referred to as PINK1^KO^) [24] or Parkin (B6.129S4-*Prkn*^*tm1Shn*^/J, hereafter referred to as Parkin^KO^) [25], as well as the recommended background control mouse strain C57Bl/6J were purchased from The Jackson Laboratory. Colonies of both the PINK1^KO^ and Parkin^KO^ were maintained in-house and housed in HEPA-filtered cages with water and food pellets, *ad libidum*. As these experiments were done concurrently with those of a previous study [13] and used the same group of C57Bl/6 control mice, the Material and Methods in the present study are largely identical to those of the earlier study except where noted.

Mice were inoculated as previously described [13] with a 1% (w/v) brain homogenate stock derived from the brains of clinically positive RML prion infected mice. The RML prion stock was titered in C57Bl/10 mice and has an infectious titer of 2 x 10^8.8^ ID_50_/g. Negative control mice were inoculated with a 1% (w/v) normal brain homogenate (NBH) stock derived from the brains of uninfected, clinically normal mice. Mice were monitored weekly for clinical signs of disease including ataxia, kyphosis, and altered grooming and nesting habits. Whole brains were collected at the clinical stage of disease and intercurrent deaths unrelated to prion infection were excluded from the final data analysis.

### Immunohistochemical analysis and lesion profile

Hematoxylin and eosin (H&E) staining and detection of PrP^Sc^ on sagittal brain sections were done as previously described [13]. PrP^Sc^ was detected using a 1:6,000 dilution of the anti-PrP rabbit monoclonal antibody EP1802Y (GeneTex, Irvine CA), which recognizes the C-terminal PrP amino acids 217–226.

Spongiform change lesion profiles in the brains of RML prion infected mice at the clinical stage of infection were assessed for 3–4 mice per strain. Nine brain regions were analyzed: frontal cortex, thalamus, hypothalamus, hippocampus, superior colliculus, midbrain, pons, medulla, and cerebellum. The extent of vacuolation was scored as follows: 0, no vacuoles; 1, scarce and unevenly scattered vacuoles that are widely distributed; 2, multiple evenly scattered vacuoles; 3, moderate number of evenly scattered vacuoles; 4, many vacuoles with some confluence; 5, dense vacuolation with a lacy appearance. The lesion profile for the C57Bl/6 control mice has been previously reported [13].

### Isolation of mitochondria and Seahorse XFe96 mitochondrial coupling assay

The isolation of brain mitochondria and mitochondrial coupling assay were done as previously described [13]. Briefly, isolated brain mitochondria from a single, clinically positive RML infected mouse or a single, NBH inoculated mice at an equivalent dpi, were analyzed on a Seahorse 96eXF assay plate (Agilent) coated with poly-D-lysine using a Seahorse XF96 Analyzer (Agilent). Mitochondrial oxygen consumption was initiated via complex II (CII) using the CII substrate succinate (10mM) and the complex I (CI) inhibitor rotenone (2μM), which were present throughout the assay. The reaction conditions were 5μg/50μl of brain mitochondria per well, 1mM ADP (saturating substrate), 1μM oligomycin (CV inhibitor), 2μM carbonyl cyanide p-trifluoromethoxyphenylhydrazone (FCCP, uncoupling reagent) and 4μM antimycin A (CIII inhibitor). These concentrations resulted in reproducible oxygen consumption rates (OCR) and respiratory control ratio (RCR) values consistent with those previously reported for healthy brain mitochondria [29]. The studies reported here were done concurrently with those of a previous paper [13] and used the same NBH and RML inoculated C57Bl/6 control mice. The calculated mitochondrial oxygen consumption rates (OCR) and respiratory control ratios (RCR) for the C57Bl/6 mice are therefore the same as those reported in the previous work [13].

### Seahorse XF Pro mitochondrial coupling assay on normal and aged mice

For PINK1^KO^, Parkin^KO^, and C57Bl/6 uninoculated, normal, younger mice (38–67 days old, one PINK1^KO^ mouse at 162 days old) and uninoculated, aged mice (256–343 days old), isolation of mitochondria and analysis of mitochondrial respiration was done as above but the mitochondrial coupling assay was done using a Seahorse XF Pro Analyzer (Agilent). Optimization of this assay for the Seahorse XF Pro Analyzer yielded slightly different parameters than those that had been used for the Seahorse XFe96 Analyzer as follows: the final concentration of oligomycin used was 1.5μM, the final concentration of FCCP used was 2.5μM, and 3 timepoints were taken for each condition measured. Isolated mitochondria from PINK1^KO^, Parkin^KO^, and C57Bl/6 mice were analyzed on the same 96-well plate to facilitate comparisons between the mouse lines.

### Calculation of OCR and RCR

For NBH and RML inoculated PINK1^KO^ and Parkin^KO^ mice, OCRs and RCRs for States 2, 3, 4o and 3u were calculated as described previously [13]. For the uninoculated normal and aged PINK1^KO^ and Parkin^KO^ mice, OCRs for States 2, 3, 4o and 3u were calculated by subtracting the average non-mitochondrial OCR from three data points from the average OCR of the three datapoints taken for each state. RCRs were calculated as for the inoculated groups. Statistics were calculated using GraphPad Prism (v9.3.1).

### Preparation of brain homogenates for Western blotting

Brains were homogenized in PBS to a final weight/volume of 20% and adjusted to a final concentration of 0.1M Tris pH 8.3, 0.1% sodium deoxycholate and 0.1% Triton X-100. Homogenates were vigorously mixed and incubated on ice for 5–10 minutes with intermittent vortexing. Pierce Universal Nuclease (ThermoFisher) was added at 0.5μl per 100μl of brain homogenate and the sample incubated for another 5 minutes. The treated lysates were spun for 1 minute at 15,000 x g and the clarified supernatant transferred to a fresh tube. For detection of PrP^Sc^, some samples were treated with proteinase K (PK) at a final concentration of 50μg/ml at 37°C for 30 minutes. Protease digestion was stopped by the addition of 0.1M phenylmethlysulfonyl fluoride (PMSF) to a final concentration of 0.01M and the samples prepared for western blot analysis as detailed below. For detection of proteins other than PrP^Sc^, samples were treated identically except that the PK and PMSF treatment steps were omitted.

### SDS-PAGE and Western blotting

For all samples, 4X NuPage lithium dodecyl sulfate (LDS) sample buffer (141mM Tris Base, 106mM Tris HCl, 2% LDS, 0.51mM EDTA, 0.22mM SERVA Blue, 0.175mM Phenol Red, 10% glycerol, 200mM DTT, final pH at 1X of 8.5) was added to a final 1X concentration and the sample heated at 95°C for 5 minutes. The sample was briefly spun, and 5μl (0.12mg brain equivalents) of the supernatant was loaded per well of a 1.0mM 26-well NuPage 4–12% Bis-Tris Midi Gel (Invitrogen). Proteins were transferred to polyvinylidene difluoride (PVDF) membrane (Millipore, Burlington MA) overnight at constant voltage (32V).

Membranes were developed using the primary antibody 6D11 (PrP residues 95–105, Biolegend, San Diego CA) at a dilution of 1:5,000 to detect PrP^Sc^. Detection of subunit proteins from mitochondrial complexes CI-CV was done using the Total OXPHOS Rodent WB Antibody Cocktail (Abcam, Waltham MA) at a dilution of 1:500. This antibody mixture recognizes key proteins in each of the complexes of the ETC: NADH:ubiquinone oxidoreductase subunit B8 (CI), succinate dehydrogenase [ubiquinone] iron-sulfur subunit B (CII), cytochrome b-c1 complex subunit 2 (CIII), cytochrome c oxidase subunit I (CIV), and ATP synthase subunit alpha (CV). As a positive control for identification of mitochondrial proteins, a single well was loaded with 2.5–5μl of a 1.5 mg/ml mitochondrial extract from rat heart tissue (Abcam, Waltham MA). Mitofusion 2 (MFN2) was detected using a rabbit monoclonal antibody at 1:2000, dynamin related protein (Drp1) was detected using a rabbit monoclonal antibody diluted 1:1000, and mouse actin was detected using a 1:10,000 dilution of a rabbit monoclonal antibody that detects both the β and γ forms of mouse actin (β/γ actin). All of these antibodies were purchased from Cell Signaling Technology, Danvers MA. All rabbit primary antibodies were detected with a 1:10,000 dilution of a goat anti-rabbit secondary antibody conjugated to alkaline phosphatase (Jackson ImmunoResearch, West Grove, PA). A goat anti-mouse antibody conjugated to alkaline phosphatase (West Grove, PA) was used at a dilution of 1:10,000 to detect 6D11 and the mitochondrial protein antibody cocktail. Alkaline phosphatase conjugates were detected using Attophos substrate (Promega). All blots were imaged on a Typhoon scanner (GE Healthcare).

### Quantitation of protein expression and statistics

Protein expression levels were quantified and analyzed as described previously [13] using the UN-SCAN-IT gel software (v7.1). Briefly, protein band intensities on digitized images were quantified by a summation of the pixels within a defined analysis region. Background correction was done by subtracting the summation of pixels in an equal sized analysis region taken from outside of the bands of interest. Relative intensities were calculated by dividing the background corrected pixel sum of protein bands of interest by the background corrected pixel sum of actin bands from the same sample. Averages, deviations, and statistical analysis were done using GraphPad Prism software (v 9.3.1).

## Supporting information

S1 Fig No spongiform change in the brains of NBH inoculated C57Bl/6, PINK1^KO^, and Parkin^KO^ mice. H&E staining for NBH inoculated C57Bl/6 (155 dpi), PINK1^KO^ (145 dpi), and Parkin^KO^ (144 dpi) mice. Representative sections from thalamus, cortex, midbrain, and cerebellum are shown. No spongiform change was observed. For all panels, scale bar = 50μm.
(PDF)

S2 Fig Lack of PrP^Sc^ deposition in C57Bl/6, PINK1^KO^,and Parkin^KO^ mice inoculated with NBH. The anti-PrP rabbit monoclonal antibody EP1802Y was used to stain for PrP^Sc^ in NBH inoculated C57Bl/6 (155 dpi), PINK1^KO^ (145 dpi), and Parkin^KO^ (144 dpi) mice. Representative sections from thalamus, cortex, midbrain, and cerebellum are shown. No PrP staining was observed. The sections are from the same mice used in S1 Fig. For all panels, scale bar = 50μm.
(PDF)

S3 Fig (A) Mitochondrial coupling assay measuring mitochondrial respiration in response to succinate for C57Bl/6 (left panel, squares and black lines), PINK1^KO^ (middle panel, squares and pink lines), and Parkin^KO^ (right panel, squares and blue lines) mice. Closed symbols and solid lines represent normal, uninoculated, young mice (n = 3; 38–67 days old with one PINK1^KO^ mouse at 162 days old) while open circles and dashed lines represent uninoculated but aged mice (n = 4; (256–343 days old). The reagents added during the assay are as described in the legend to Fig 7. (B) OCRs and (C) RCRs for the data in Panel A. Data were calculated as described in the Materials and Methods. Black bars = C57BL/6, pink bars = PINK1^KO^, blue bars = Parkin^KO^. Solid bars represent uninoculated young mice while hatched bars represent uninoculated aged mice. Mean + SEM is shown. Statistical analysis was done using a 1-way ANOVA with Dunnett’s post-test with C57Bl/6 samples set as the control. *p value = 0.033–0.044; **p value = 0.0045; ***p value = 0.0002.
(PDF)

S1 Raw images Raw images of western blots for Figs 5, 6 and 8. (PDF)
