# Supplementary figures and images for: The PINK1/Parkin pathway of mitophagy exerts a protective effect during prion disease
Source: PLoS One. 2024 Feb 23;19(2):e0298095. doi: 10.1371/journal.pone.0298095 (PMC10889866; doi:10.1371/journal.pone.0298095)

Thalamus

Cortex

Midbrain

Cerebellum

C57Bl/6

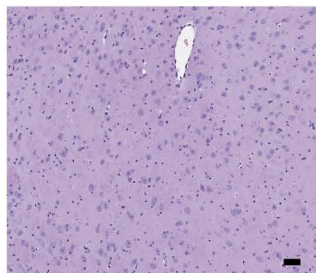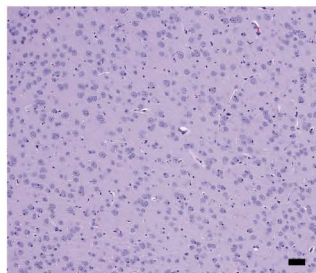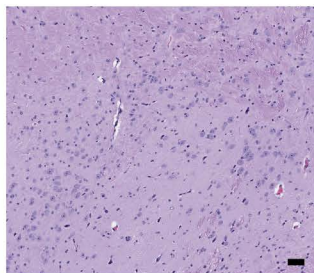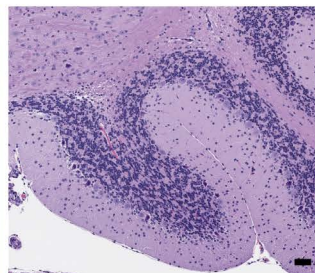PINK1<sup>KO</sup>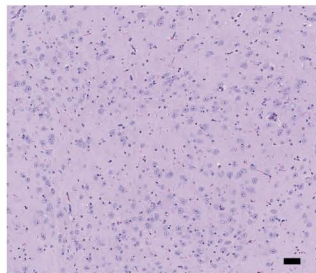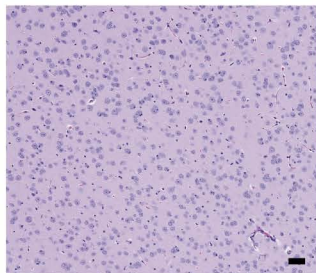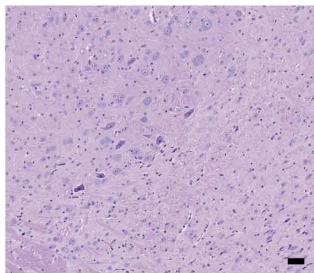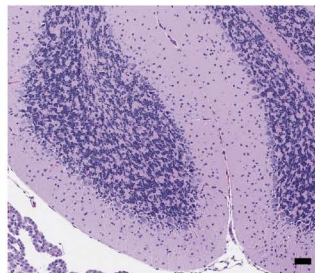Parkin<sup>KO</sup>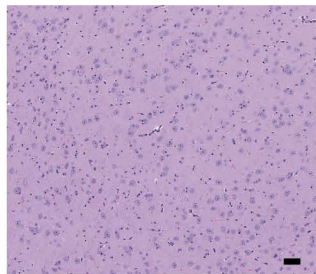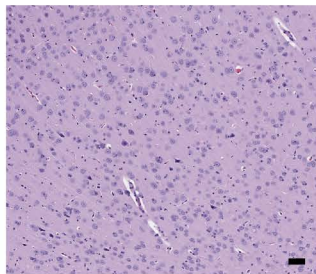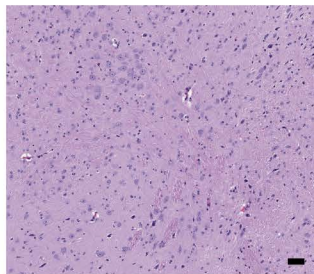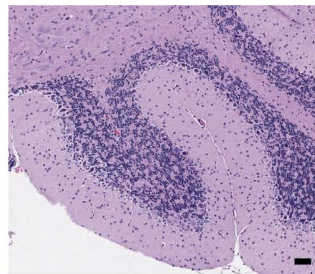

Supplement: S1 Fig — H&E staining for NBH inoculated C57Bl/6 (155 dpi), PINK1KO (145 dpi), and ParkinKO (144 dpi) mice. Representative sections from thalamus, cortex, midbrain, and cerebellum are shown. No spongiform change was observed. For all panels, scale bar = 50μm. (PDF) [file pone.0298095.s001.pdf]

Thalamus

Cortex

Midbrain

Cerebellum

C57Bl/6

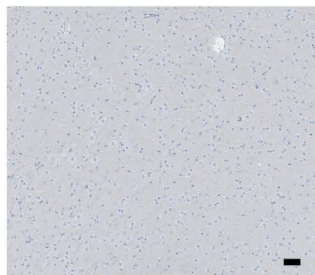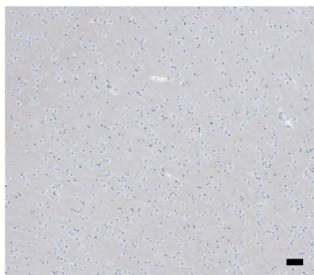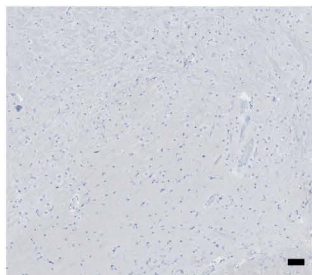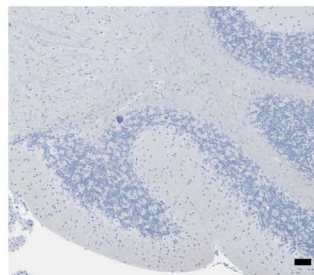PINK1<sup>KO</sup>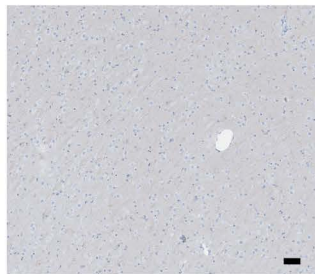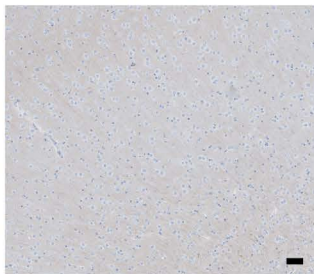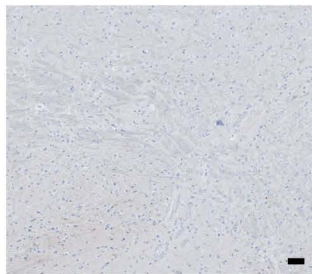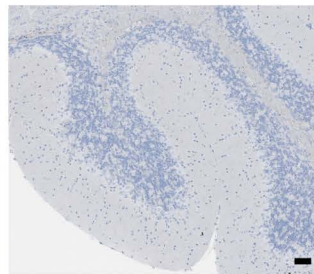Parkin<sup>KO</sup>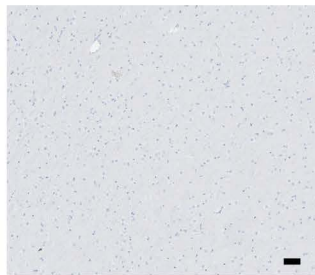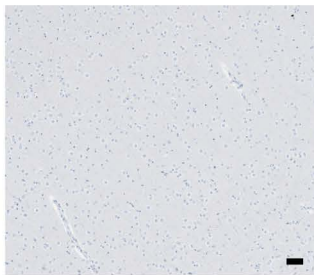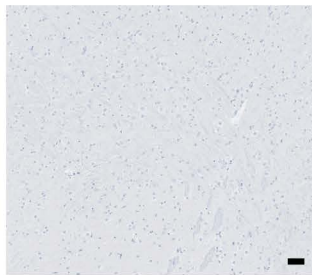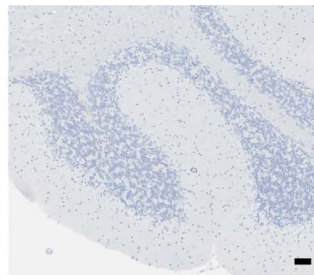

Supplement: S2 Fig — The anti-PrP rabbit monoclonal antibody EP1802Y was used to stain for PrPSc in NBH inoculated C57Bl/6 (155 dpi), PINK1KO (145 dpi), and ParkinKO (144 dpi) mice. Representative sections from thalamus, cortex, midbrain, and cerebellum are shown. No PrP staining was observed. The sections are from the same mice used in S1 Fig. For all panels, scale bar = 50μm. (PDF) [file pone.0298095.s002.pdf]

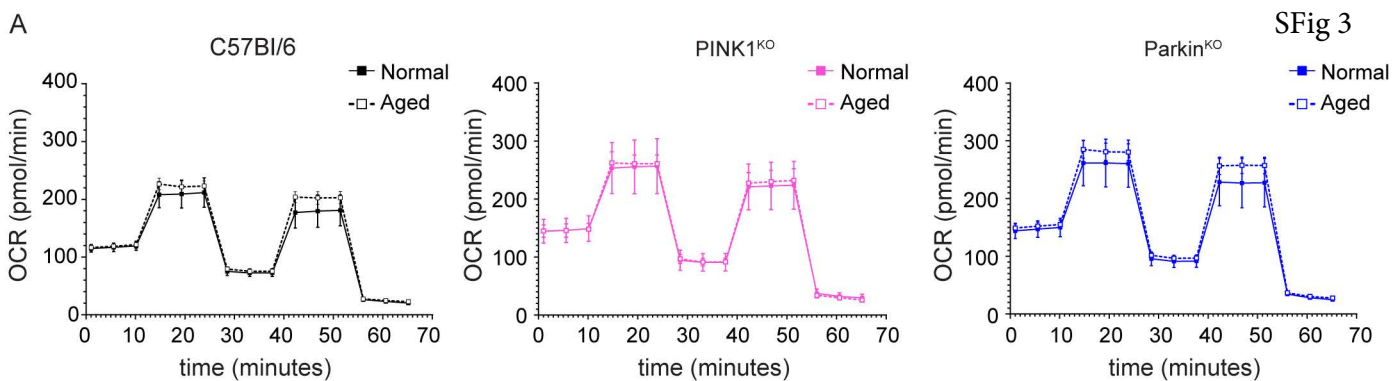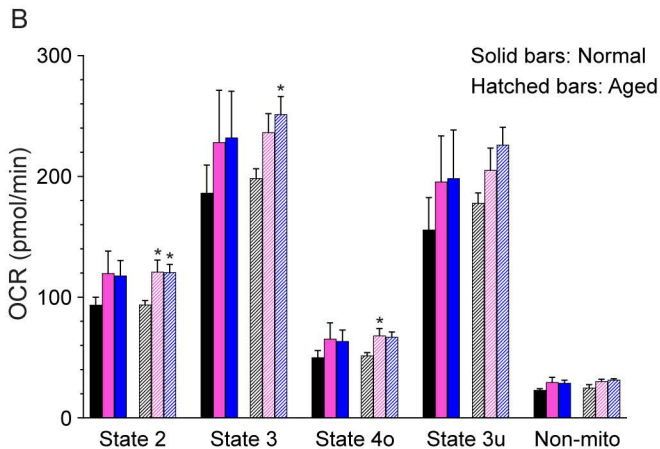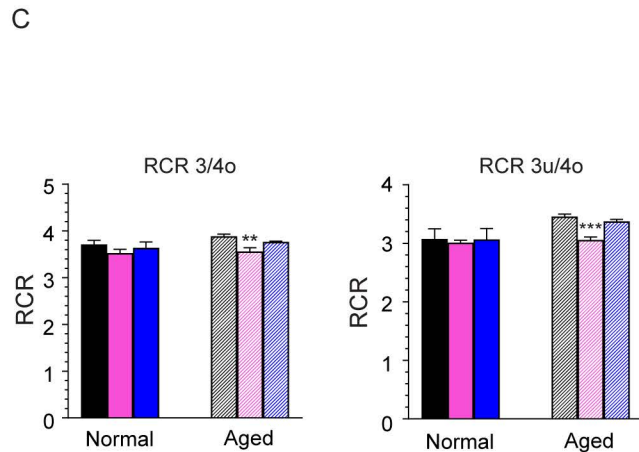

Supplement: S3 Fig — (A) Mitochondrial coupling assay measuring mitochondrial respiration in response to succinate for C57Bl/6 (left panel, squares and black lines), PINK1KO (middle panel, squares and pink lines), and ParkinKO (right panel, squares and blue lines) mice. Closed symbols and solid lines represent normal, uninoculated, young mice (n = 3; 38–67 days old with one PINK1KO mouse at 162 days old) while open circles and dashed lines represent uninoculated but aged mice (n = 4; (256–343 days old). The reagents added during the assay are as described in the legend to Fig 7. (B) OCRs and (C) RCRs for the data in Panel A. Data were calculated as described in the Materials and Methods. Black bars = C57BL/6, pink bars = PINK1KO, blue bars = ParkinKO. Solid bars represent uninoculated young mice while hatched bars represent uninoculated aged mice. Mean + SEM is shown. Statistical analysis was done using a 1-way ANOVA with Dunnett’s post-test with C57Bl/6 samples set as the control. *p value = 0.033–0.044; **p value = 0.0045; ***p value = 0.0002. (PDF) [file pone.0298095.s003.pdf]
