# Supplementary material for: The PINK1/Parkin pathway of mitophagy exerts a protective effect during prion disease
Source: PLoS One. 2024 Feb 23;19(2):e0298095. doi: 10.1371/journal.pone.0298095 (PMC10889866; doi:10.1371/journal.pone.0298095)
Supplement: S1 Raw images — (PDF) [file pone.0298095.s004.pdf]

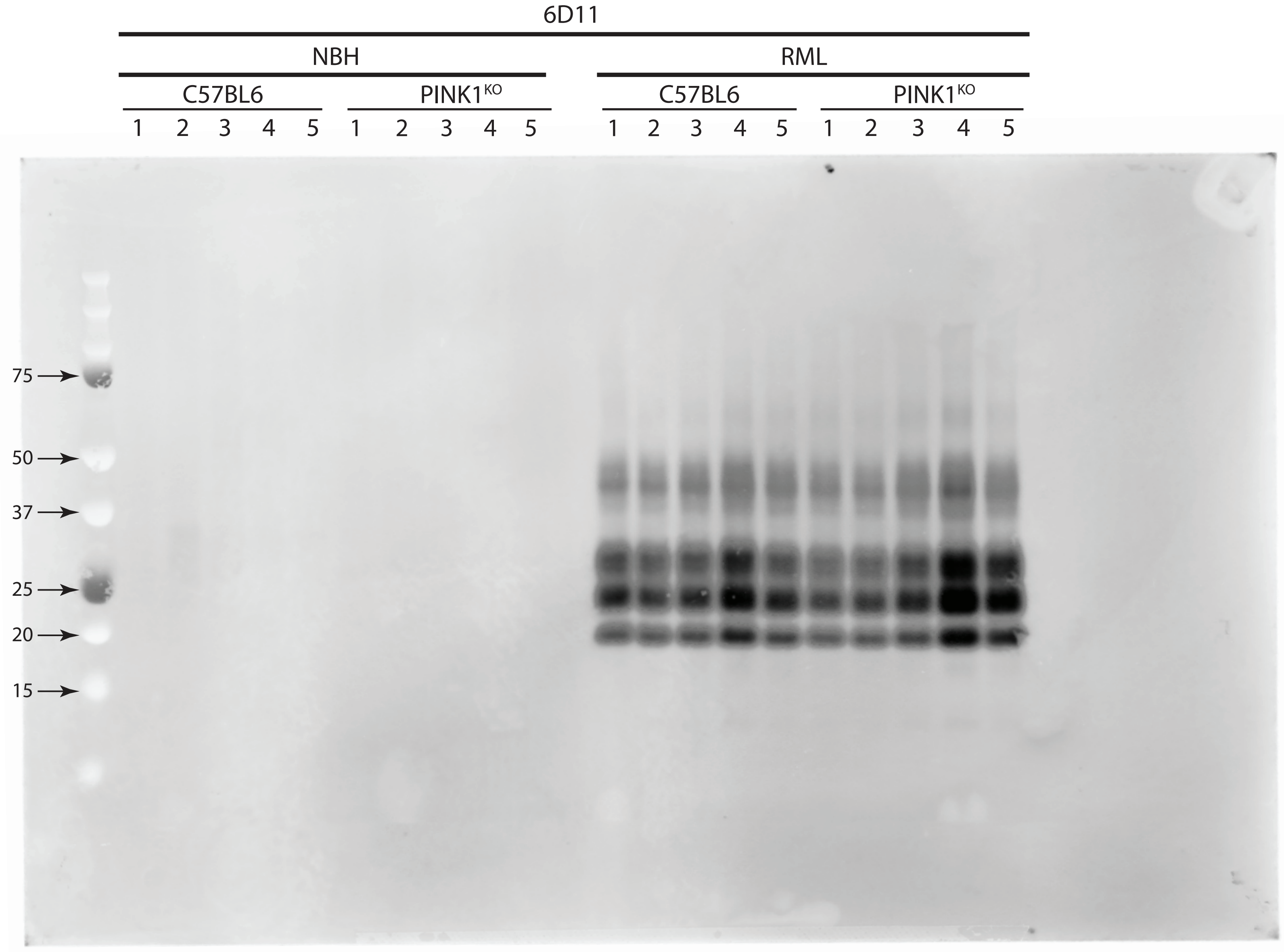
6D11
NBH
RML
C57BL6
PINK1KO
C57BL6
PINK1KO
1 2 3 4 5 1 2 3 4 5 1 2 3 4 5 1 2 3 4 5
75
50
37
25
20
15

Figure 5A

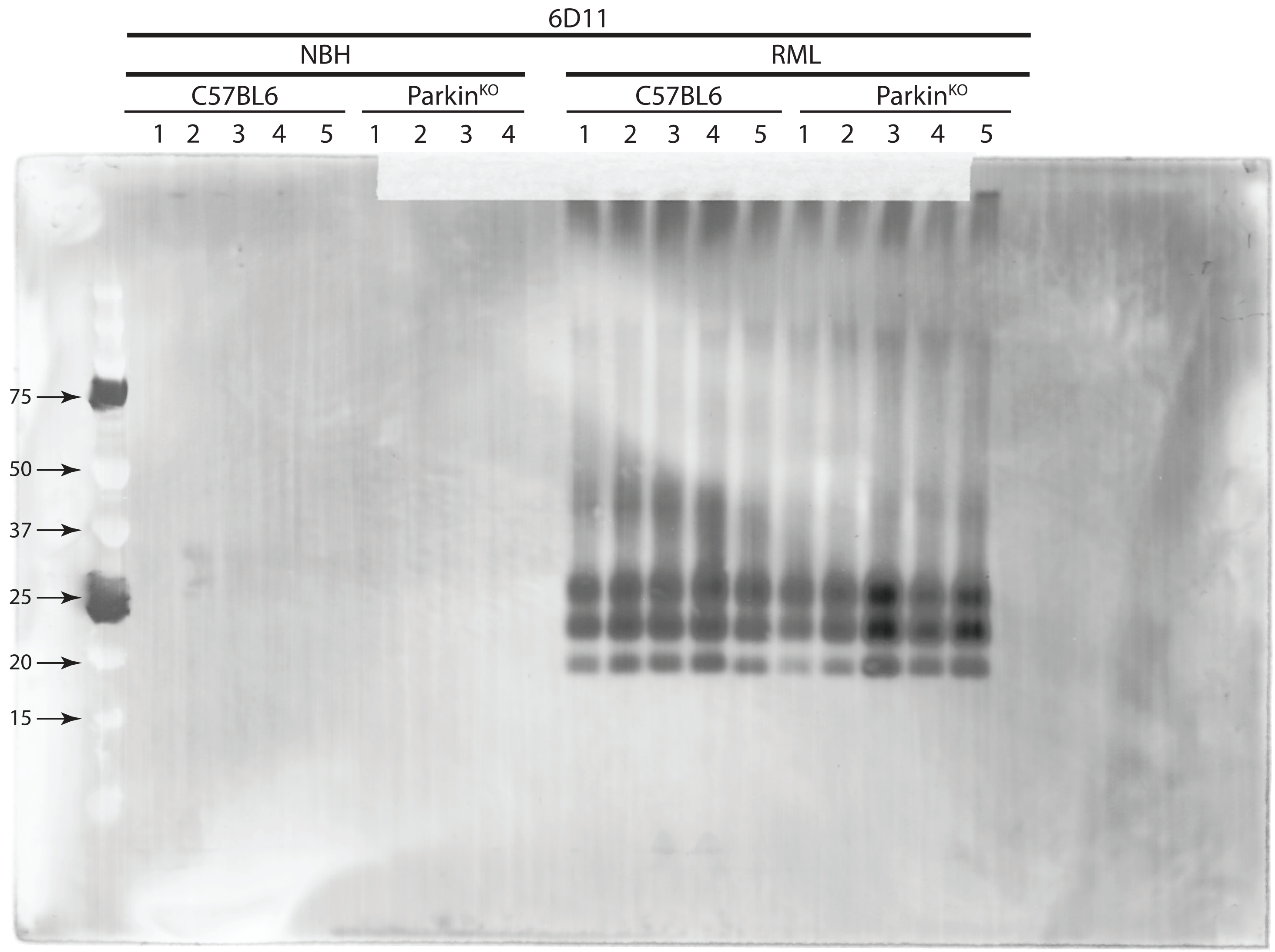
6D11
NBH
RML
C57BL6
ParkinKO
C57BL6
ParkinKO
1 2 3 4 5 1 2 3 4
1 2 3 4 5 1 2 3 4 5
75
50
37
25
20
15

Figure 5B

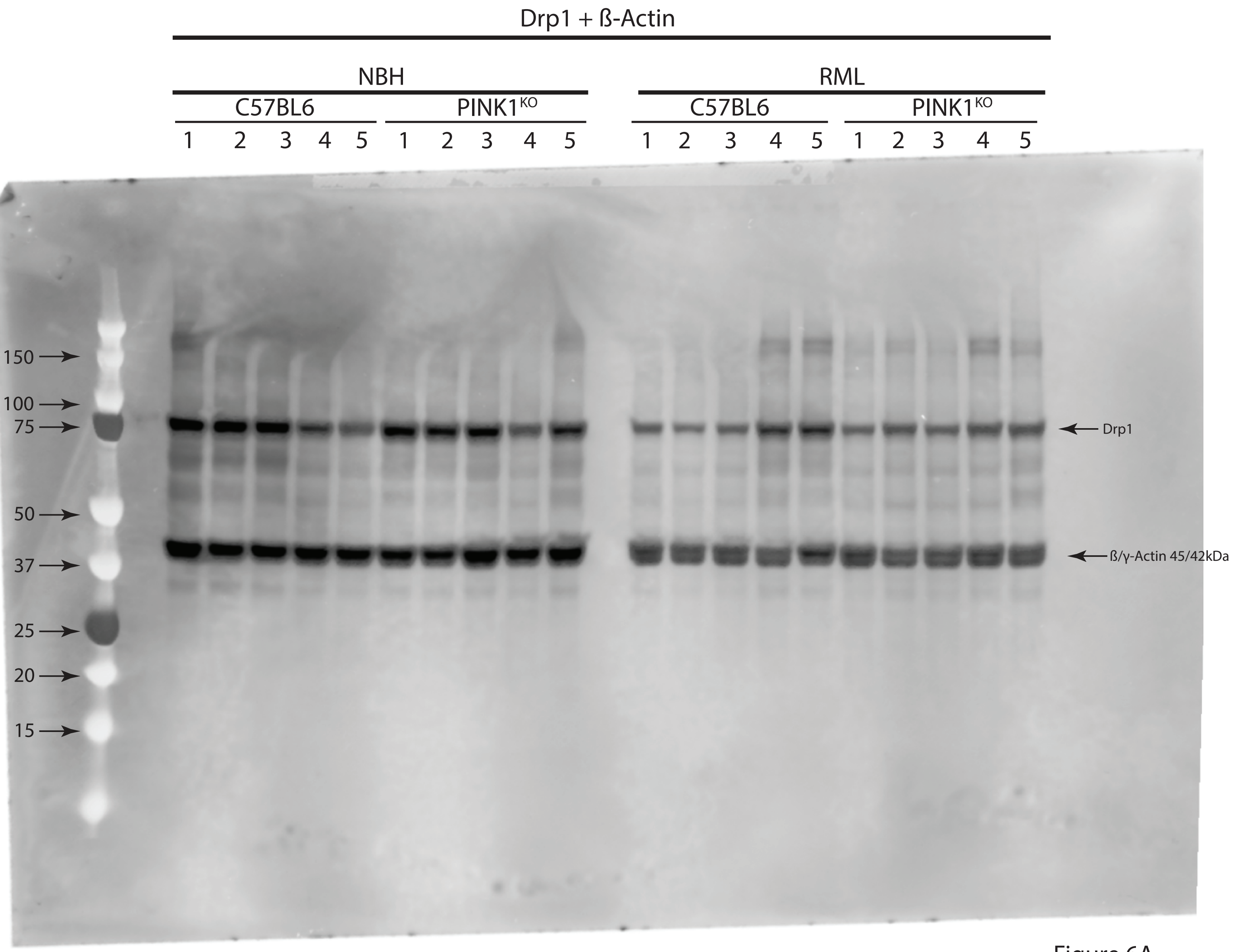

Figure 6A
Top

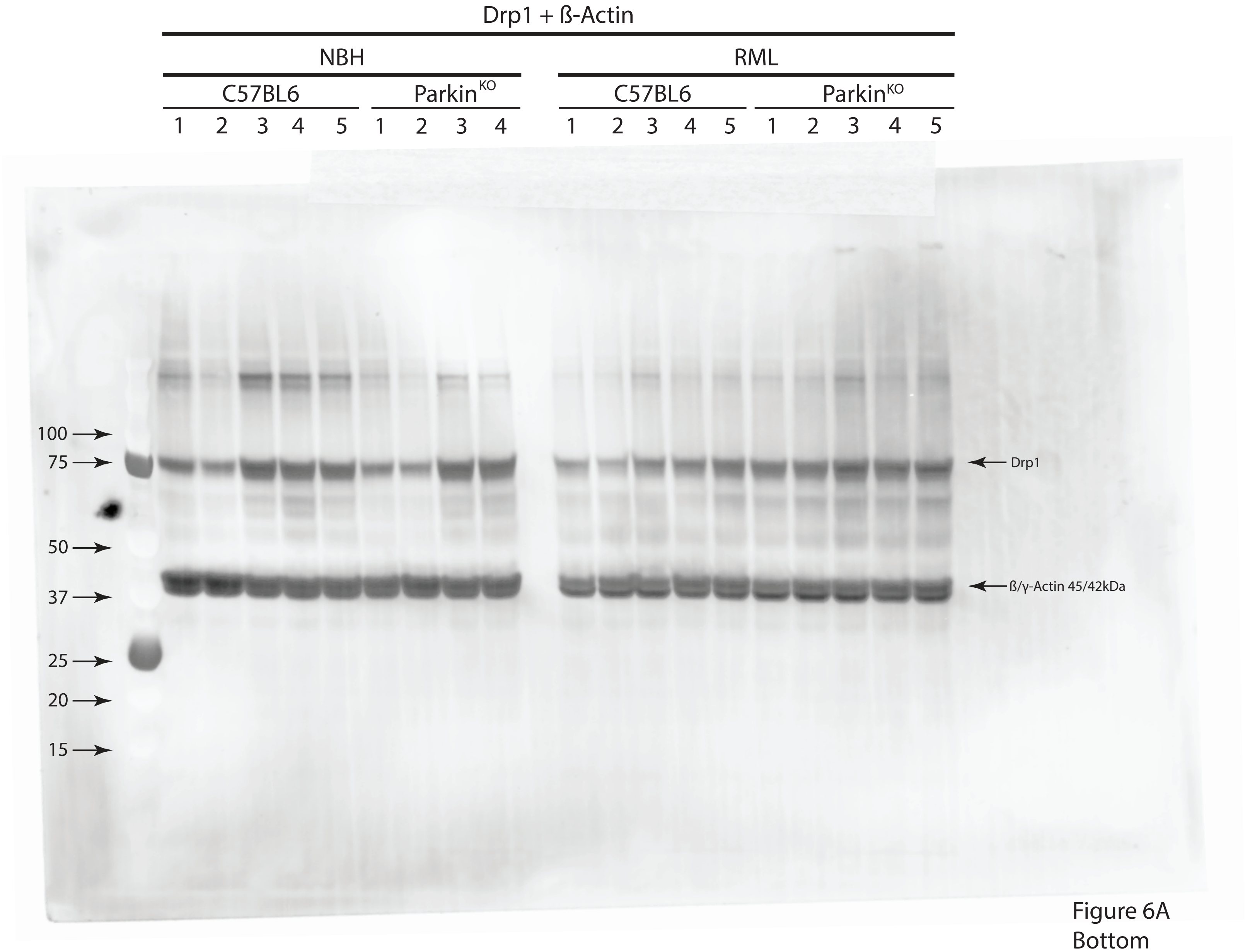

Figure 6A
Bottom

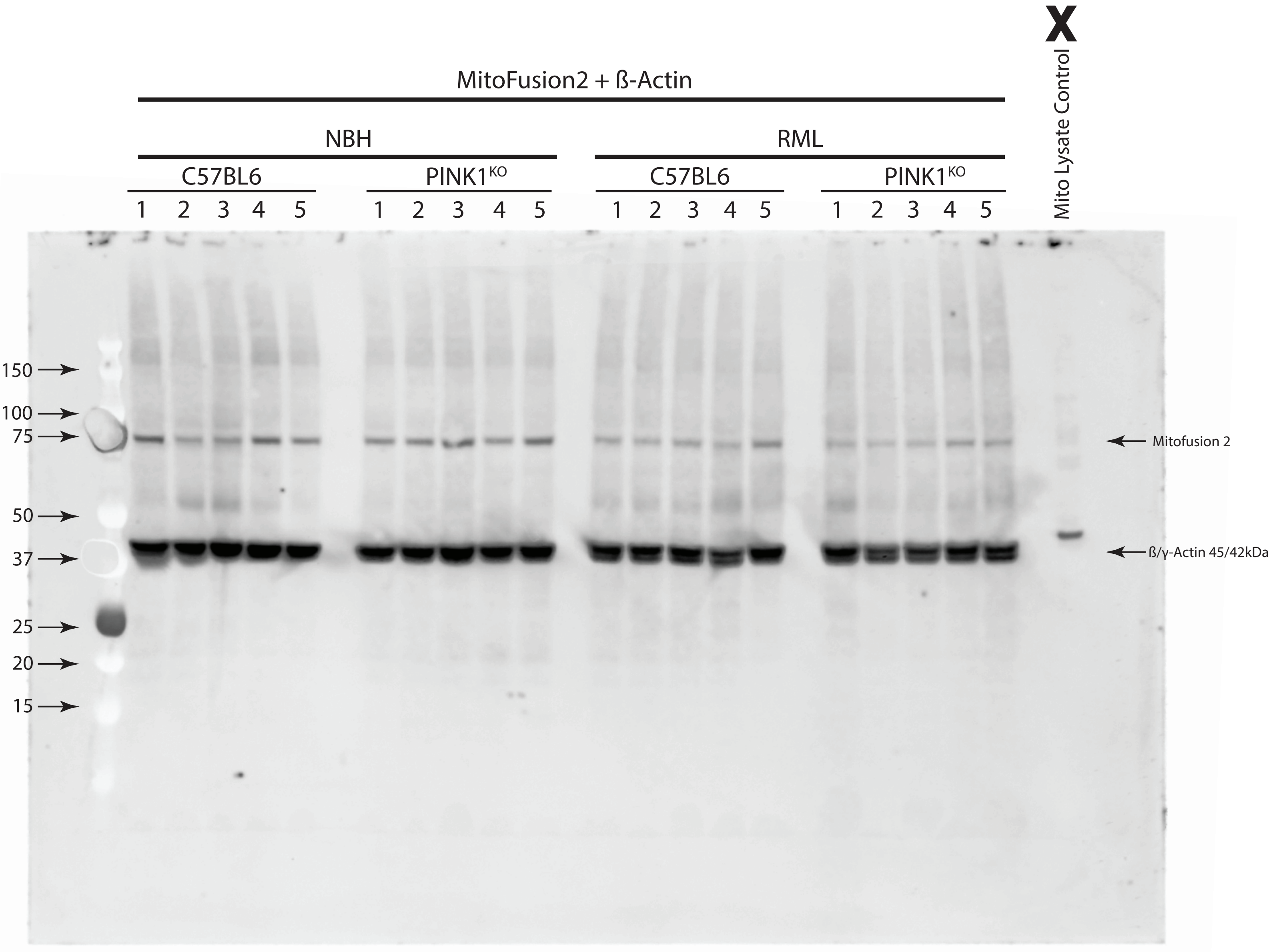

Figure 6B
Top

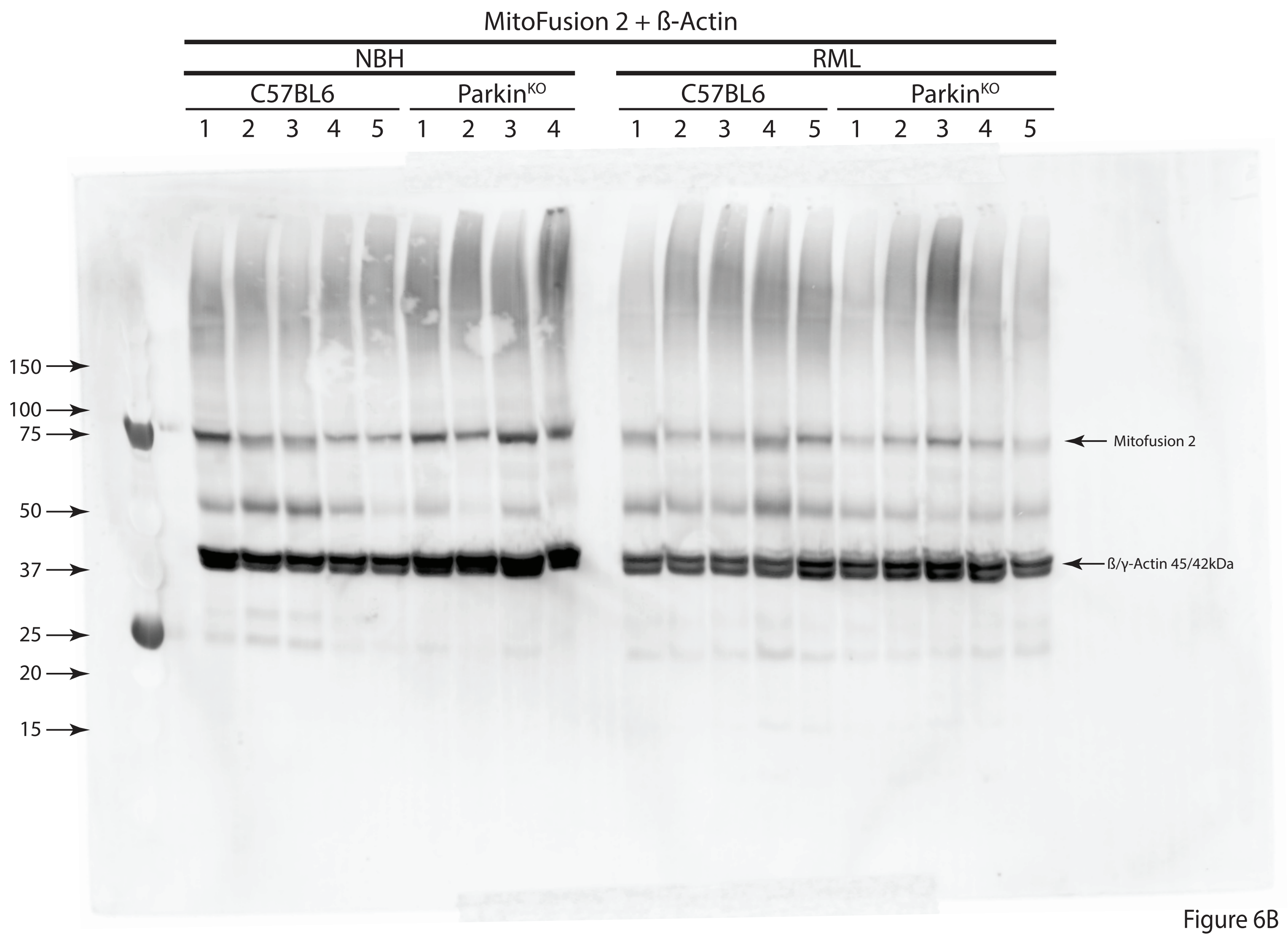

Figure 6B
Bottom

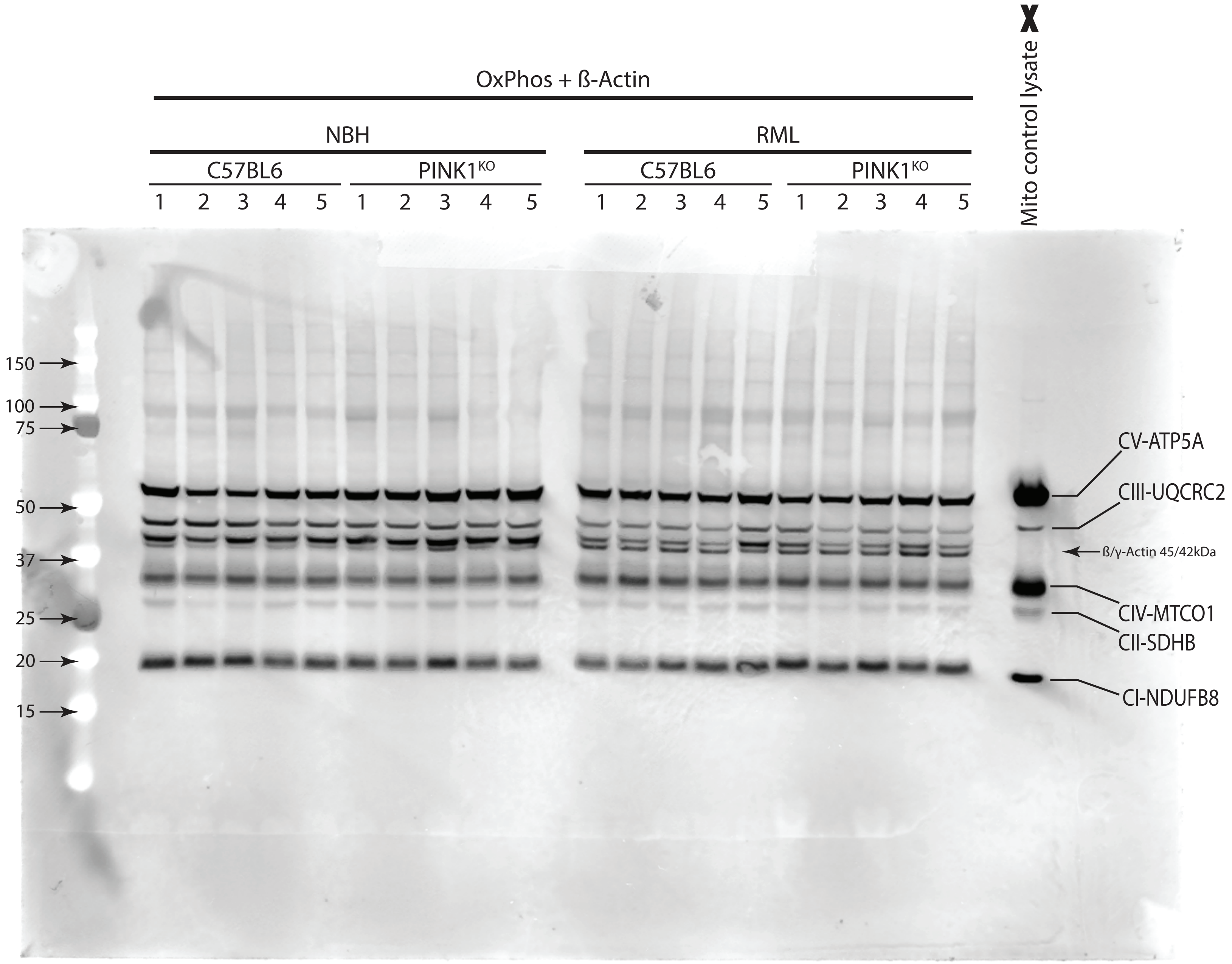

OxPhos + ß-Actin
NBH
RML
C57BL6
PINK1KO
C57BL6
PINK1KO
1 2 3 4 5 1 2 3 4 5 1 2 3 4 5 1 2 3 4 5
Mito control lysate
150
100
75
50
37
25
20
15
CV-ATP5A
CIII-UQCRC2
ß/γ-Actin 45/42kDa
CIV-MTCO1
CII-SDHB
CI-NDUFB8

Figure 8A

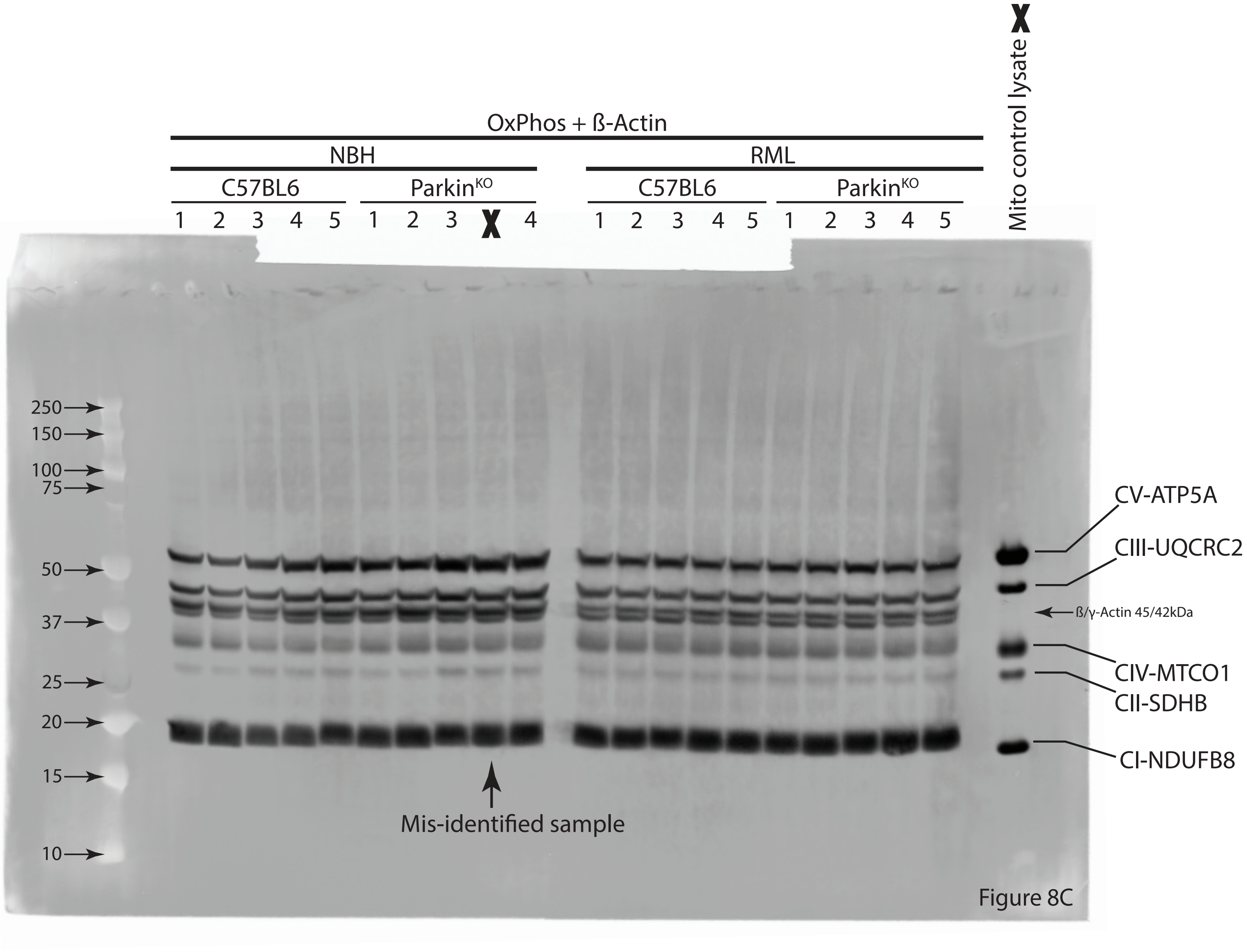

OxPhos + ß-Actin
NBH
RML
C57BL6
ParkinKO
C57BL6
ParkinKO
1 2 3 4 5 1 2 3 X 4
1 2 3 4 5 1 2 3 4 5
Mito control lysate
X
250
150
100
75
50
37
25
20
15
10
CV-ATP5A
CIII-UQCRC2
ß/γ-Actin 45/42kDa
CIV-MTCO1
CII-SDHB
CI-NDUFB8
Mis-identified sample

Figure 8C
